# Novel potential pharmacological applications of dimethyl fumarate—an overview and update

**DOI:** 10.3389/fphar.2023.1264842

**Published:** 2023-09-07

**Authors:** Giorgia Bresciani, Federico Manai, Sergio Davinelli, Paolo Tucci, Luciano Saso, Marialaura Amadio

**Affiliations:** ^1^ Section of Pharmacology, Department of Drug Sciences, University of Pavia, Pavia, Italy; ^2^ Department of Biology and Biotechnology L. Spallanzani, University of Pavia, Pavia, Italy; ^3^ Department of Medicine and Health Sciences “V. Tiberio”, University of Molise, Campobasso, Italy; ^4^ Department of Clinical and Experimental Medicine, University of Foggia, Foggia, Italy; ^5^ Department of Physiology and Pharmacology Vittorio Erspamer, Sapienza University, Rome, Italy

**Keywords:** Nrf2 pathway, drug repurposing, dimethyl fumarate, inflammation, oxidative stress, antioxidant, cardiovascular, neurodegenerative

## Abstract

Dimethyl fumarate (DMF) is an FDA-approved drug for the treatment of psoriasis and multiple sclerosis. DMF is known to stabilize the transcription factor Nrf2, which in turn induces the expression of antioxidant response element genes. It has also been shown that DMF influences autophagy and participates in the transcriptional control of inflammatory factors by inhibiting NF-κB and its downstream targets. DMF is receiving increasing attention for its potential to be repurposed for several diseases. This versatile molecule is indeed able to exert beneficial effects on different medical conditions through a pleiotropic mechanism, in virtue of its antioxidant, immunomodulatory, neuroprotective, anti-inflammatory, and anti-proliferative effects. A growing number of preclinical and clinical studies show that DMF may have important therapeutic implications for chronic diseases, such as cardiovascular and respiratory pathologies, cancer, eye disorders, neurodegenerative conditions, and systemic or organ specific inflammatory and immune-mediated diseases. This comprehensive review summarizes and highlights the plethora of DMF’s beneficial effects and underlines its repurposing opportunities in a variety of clinical conditions.

## 1 Introduction

Dimethyl fumarate (DMF) is a methyl ester of fumaric acid (FA) which has recently become the object of a renewed growing interest in pharmacological research, due to its antioxidant, anti-inflammatory, neuroprotective and anti-proliferative activities, together with its favorable safety and tolerability profiles.

The history of DMF’s applications is a curious one, dating back to the beginning of the 20th century, when it was used in industrial settings as fungicide and desiccant in the shipping of sofas. The first mention of DMF in a medical context was linked to eczematous burns in subjects who had taken contact with “poison chairs” (Ropper, 2012). Its repurposing as a pharmakon began in the 1950s, when a German biochemist suffering from *Psoriasis vulgaris* administered it to himself (Schweckendiek, 1959), erroneously believing the disease was due to a Krebs cycle metabolic dysfunction and fumarate deficiency. This episode marked a substantial shift in both use and reputation of DMF: from skin *toxic* to potential skin *medicine*. The first DMF-containing drug was approved for the treatment of severe psoriasis in 1994, in Germany, with the trade name Fumaderm^®^, a combination of 60% DMF and three salts of monoethyl fumarate (MEF) for systemic use; in 2008, the clinical indication of Fumaderm^®^ was expanded to include moderate psoriasis (Mrowietz et al., 2016).

Since then, the off-label use of DMF and other FA esters (FAEs) for psoriasis took place in several European Countries beside Germany (Landeck et al., 2018) and gave impulse to a better characterization of the efficacy profile of FAEs and DMF, the main active component. DMF’s anti-inflammatory properties, along with the tolerable safety profile seen in the long-term management of psoriasis (Reich et al., 2009), sparked renewed interest in the drug’s potential as a therapeutic strategy for other immune-mediated diseases, leading to the evaluation of its clinical use in multiple sclerosis (MS) (Kappos et al., 2008; Fox et al., 2012; Gold et al., 2012; Meissner et al., 2012). In 2013, DMF was marketed under the trade name Tecfidera^®^ (BG-12) and approved as the first-line therapy for relapsing–remitting MS (RRMS) in patients from 13 years age in Europe and US (European Medicines Agency, 2013; Food and Drug Administration, 2013). A further advance in the use and popularity of DMF across Europe dates to 2017, when the EMA approved Skilarence^®^ (European Medicines Agency, 2017), a new oral formulation of the sole DMF for the treatment of moderate-to-severe chronic plaque psoriasis in adult patients.

DMF is still the gold standard in the treatment of plaque psoriasis and a good therapeutic option for RRMS; as understanding of the pathogenesis of these diseases improved, DMF beneficial effects have been more correctly attributed to its modulation of the immune and inflammatory responses (Miljković et al., 2015; Landeck et al., 2018). However, increasing evidence indicates that DMF effects are pleiotropic, and its clinical use may be broader than was conceived for decades. Accordingly, in recent years, several studies have underlined numerous promising alternatives for the repurposing of DMF, as its antioxidant, anti-inflammatory, neuroprotective properties constitute a convincing rationale for DMF use in different diseases (for comprehensive reviews on the specific topics, see, for example, Tastan et al., 2022; Manai et al., 2022; Morris et al., 2022; Sánchez-García et al., 2021; Kourakis et al., 2020; Al-Ani et al., 2020; Sachinvala et al., 2020; Scuderi et al., 2020; Saidu et al., 2019).

After a brief description of DMF’s mechanisms of actions and cellular/molecular pathways underlying its biological effects, the present review provides an exhaustive analysis of the most recent literature evidence for DMF novel pharmacological applications by considering both preclinical and clinical data.

## 2 Mechanisms of action: molecular targets and pathways

As an α,β-unsaturated carboxylic ester, DMF (C_6_H_8_O_4_; MW: 144.13 g/mol), exhibits a high degree of electrophilicity. Upon oral administration, the compound undergoes initial metabolism via esterase, thereby yielding its primary active metabolite, monomethyl fumarate (MMF) (Werdenberg et al., 2003). DMF, MMF and other fumarates interact with the antioxidant glutathione (GSH), through a Michael addition reaction with cysteine residues; on the other hand, enzymes in the cytochrome P450 complex are not involved in DMF metabolism (Aubets et al., 2019).

To maximize DMF absorption in the small intestine, in clinical practice orally administered formulations are characterized by a delayed release (Kappos et al., 2008). DMF detection in systemic circulation is not significant, and less than 0.1% of the applied DMF dosage is detected in urine (Litjens et al., 2004). There is a lack of evidence pertaining to the accumulation of DMF subsequent to the administration of multiple doses (Sheikh et al., 2013). Excretion of DMF through urine and faeces is also negligible since it is mostly eliminated through CO_2_ exhalation.

MMF systemic peak concentration, which is usually reached 2–2.5 h after oral administration, is delayed by concomitant administration of fatty foods; however, this does not influence the area under the curve, which is proportional to the administered dose (Linker and Haghikia, 2016), instead helping to manage the intensity of the adverse effects affecting the gastrointestinal (GI) tract (Paolinelli et al., 2022).

**FIGURE 1:**
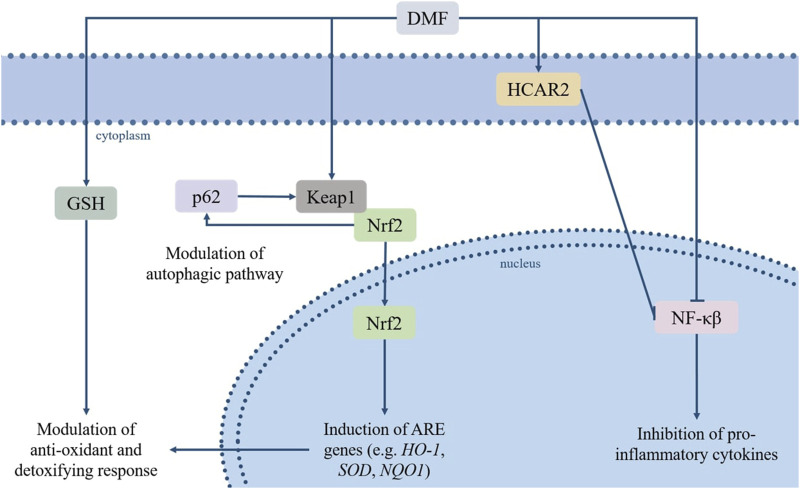
DMF’s immunomodulatory and antioxidative effects rely on its regulation of several pathways, among which the Keap1/Nrf2/ARE and NF-κB pathways, the modulation of GSH levels, its agonism of HCAR2 and its effects on the autophagic system. Based on these properties, DMF has sparked interest due to its potential repurposing for a variety of pathologies characterized or aggravated by inflammatory processes and oxidative stress. Abbreviations: dimethyl fumarate (DMF), glutathione (GSH), Hydroxycarboxylic Acid Receptor 2 (HCAR2), sequestrome 1 (p62/SQSTM1), Kelch-like ECH-associated protein (Keap1), nuclear factor erythroid 2-related factor 2 (Nrf2), nuclear factor kappa-light-chain-enhancer of activated B cells (NF-κB), *hemoxygenase-1* (*HO1*), *quinoline oxidoreductase-1* (*NQO1*), *superoxide dismutase* (*SOD*).

The number of pathways involved in the biological activities of DMF, and their relative contribution to its clinical effects, are not entirely understood, as the pharmacodynamics of FAEs are still to be completely elucidated. Moreover, the exact impact of DMF on each pathway is hard to determine due to several variables (e.g., the level of tissue exposure, the rate of absorption and metabolism); however, some main cellular targets/pathways relevant to its therapeutic effects have been established, and include the nuclear factor erythroid 2-related factor 2 (Nrf2) transcription pathway, the nuclear factor kappa-light-chain-enhancer of activated B cells (NF-κB) pathway, glutathione and its biosynthesis pathway, the autophagy system, and the hydroxycarboxylic acid receptor 2 (HCAR2) (Figure 1).

### 2.1 Nrf2/ARE pathway

Oxidative stress is a state of imbalance between cellular oxidants and antioxidants, in which the oxidant agents induce a disruption in redox signaling and trigger a cascade leading to several forms of cellular damage. Oxidants and reactive oxygen species can generate and accumulate in all types of organelles, and this process plays a key role in the pathogenesis of a variety of diseases (Azzi, 2022). The Nrf2 pathway is an essential protective mechanism against oxidative damage, exerting its cytoprotective action through the regulation of a wide array of antioxidant response elements (AREs)-bearing genes (Zhao et al., 2017), and of genes controlling fundamental cellular functions and processes (e.g., cell survival and proliferation, autophagy, regulation of drug elimination, DNA repair and mitochondrial function) (Hayes and Dinkova, 2014; Rojo de la Vega et al., 2018).

Under physiological conditions, the Kelch-like ECH-associated protein (Keap1) maintains Nrf2 sequestered and inactive in the cytoplasm and induces its degradation via proteasome. The cysteine residues-containing domains within the Keap1 structure work as a redox sensor, allowing it to register changes in the levels of oxidant or electrophilic agents and to respond accordingly, destabilizing the Nrf2/Keap1 complex, leading to translocation of Nrf2 to the nucleus and preventing its proteolysis (Kobayashi et al., 2004). DMF, an electrophilic agent, causes the oxidation of the reactive thiols in the Keap1 protein, and thus induces Nfr2 nuclear translocation and activation (Linker et al., 2011). Among Nrf2-regulated genes are *hemoxygenase-1* (*HO1*) and *quinoline oxidoreductase-1* (*NQO1*), particularly relevant for their antioxidant effects, and genes involved in the expression of xenobiotic metabolism enzymes and drug efflux pumps (Scannevin et al., 2012; Zhou et al., 2013). Moreover, Nrf2 promotes the activation of GSH biosynthesis pathway, directing intracellular cysteine toward GSH synthesis by modulating glutamate-cysteine ligase (GCL) and glutathione synthase (GSS) A complex crosstalk between Nrf2 and different components of the autophagic system has also been observed; deficiencies in autophagy-related proteins may lead to the persistent activation of Nrf2 through the binding of p62 to Keap1, which isolates Keap1 and leads to Nrf2 activation (Inami et al., 2011; Ni et al., 2012; Ni et al., 2014). The interaction between the two factors works both ways, as p62 is also a target gene positively regulated by Nrf2 (Jain et al., 2010). A fundamental role in the p62-dependent sequestration of Keap1, and the resulting activation of the Nrf2 pathway, is played by the mammalian target of rapamycin complex 1 (mTORC1). It is the phosphorylation of p62 at Ser349, which can be carried out by mTORC1in response to conditions of oxidative stress, nutrient stress, damaged mitochondria or invading pathogens, that induces Keap1 sequestration (Duran et al., 2011; Ichimura et al., 2013).

In conclusion, Nrf2 represents a pharmacological target fully embraced by both classic and systems medicine approaches for drug development and drug repurposing, since alterations of Nrf2 signaling are identified as a common molecular mechanism in a network of different pathophenotypes comprised in the so-called “Nrf2 diseasome” (Cuadrado et al., 2018).

### 2.2 NF-κB

The NF-κB protein complex includes five different subunits (i.e., RelA/p65, RelB, c-Rel, NF-κB1 p50, NF-κB2 p52) and functions as a transcription factor, controlling the expression of genes involved in immunity and inflammation, the development of lymphoid organs and the maturation of B-cells (Hayden and Ghosh, 2008). The phosphorylation and acetylation of the RelA/p65 subunit is essential for the activation of the whole complex; again, as an electrophilic compound, DMF reacts with the cysteine residues on RelA/p65 and triggers a covalent modification, preventing the phosphorylation of NF-κB and its translocation to the nucleus (Kastrati et al., 2016). This inhibitory effect on NF-κB results in a rebalancing between pro- and anti-inflammatory factors and triggers both alterations in the maturation of antigen-presenting cells and a switch in T helper cells subtype - from type 1 and 17 to type 2 - which in turn leads to a shift in cytokines production in favor of interleukin 4 and 5 (Peng et al., 2012; McGuire et al., 2016).

### 2.3 Glutathione

Glutathione (GSH) functions as the main antioxidant and detoxifying system in cells, granting a protective mechanism against any damage connected to increase in oxidant agents, which represent a key factor in the pathogenesis of several diseases. As previously mentioned, by Nrf2 activation, DMF regulates the gene expression of enzymes belonging to the GSH biosynthetic pathway. In addition, a physical interaction between DMF and GSH may occur and affect GSH availability and function. More specifically, DMF interacts with the thiol groups on the GSH molecule, which leads to a Michael addition reaction and consequent modifications in GSH levels and function. This initial sequestration of GSH by DMF may trigger an adaptive response, inducing a subsequent increase in both GSH production and recycling (Hoffmann et al., 2017). This latter DMF-induced upregulation of GSH levels contributes in turn to the inhibition of NF-κB and, subsequently, of inflammatory cytokines expression, independently from the Nrf2 pathway activation (Gillard et al., 2015). However, these effects are dose-dependent and also vary based on the cell type; low concentrations of DMF in nontumorigenic cells upregulate GSH levels as mentioned, while DMF administered to tumorigenic cells triggers GSH depletion through the inhibition of the first enzyme in its biosynthetic pathway, γ-GCS (Saidu et al., 2017). This latter dose-dependent effect may represent a useful strategy in cancerous cells, where treatment with high dosage of DMF results in depletion of intracellular GSH and increased ROS levels and, ultimately, cell death. On the other hand, the DMF-induced GSH decrease can also lead to undesired apoptotic death in some noncancerous cell types, such as T-cell lymphocytes (Treumer et al., 2003). Lymphopenia is in fact one of the most troubling adverse effects of DMF, although it occurs in a minority of patients and is likely due, at least in part, to genetic predisposition (Treumer et al., 2003; Paolinelli et al., 2022).

### 2.4 Autophagy

Autophagy plays a critical role in the homeostasis of the cell and its functions, eliminating damaged organelles and other superfluous or dysfunctional intracellular components, and contributing to cell survival against stress conditions or specific treatment. This makes the autophagic pathway a promising target in a variety of conditions, with pharmacological modulation aiming either to stimulate autophagy and to exploit its protective role, or to inhibit it from constituting an escape mechanism, like in cancer cells. DMF’s interaction with the autophagic pathway is mainly dependent on DMF-mediated activation of Nrf2, as the two pathways are correlated by a positive feedback loop: Nrf2 activation can result in the transcription of *TP53*, an autophagy inducer, whereas the autophagy adaptor protein SQSTM1/p62 may induce the nuclear translocation of Nrf2 through the autophagy-dependent degradation of Keap1 (Hu et al., 2022).

### 2.5 Additional pathways

Beside the upper mentioned pathways, the anti-inflammatory activity exerted by DMF is also reliant on its agonism of the hydroxycarboxylic acid receptor 2 (HCAR2), as demonstrated *in vivo* in a mouse model of experimental autoimmune encephalomyelitis (EAE) (Chen et al., 2014). HCAR2, expressed in several cell types ranging from macrophages and neutrophils to intestinal or retinal epithelial cells, is implicated in anti-inflammatory responses (Offermanns, 2017). This HCAR2-related anti-inflammatory activity is dependent on a decrease in immune cells infiltration, likely resulting from HCAR2 interference with neutrophil adhesion to endothelial cells and chemotaxis. Blockade of HCAR2 completely reverses the effect of DMF on the expression of inflammatory and oxidative stress-related molecules (Parodi et al., 2015), demonstrating the involvement of HCAR2 in its clinical activity. The HCAR2-dependent regulation of the inflammatory response is likely due to activation of the G protein signalling cascade, which results in AMPK activation and increased NAD^+^-dependent protein deacetylase sirtuin-1 (SIRT-1) levels; SIRT-1 directly inhibits NF-κB through the deacetylation of the RelA/p65 subunit (Yeung et al., 2004), leading to previously discussed anti-inflammatory downstream effects. On the other hand, the pleiotropic nature of HCAR2 leads to different downstream outcomes, depending on cell type and experimental conditions; this pleiotropic effect of HCAR2 activation is behind some of the side effects of DMF treatment (e.g., flushing and gastrointestinal symptoms). Activation of HCAR2 by MMF has been demonstrated to induce cyclooxygenase-2 (COX-2)-mediated synthesis of prostaglandins in keratinocytes and Langerhans cells, leading to flushing, which occurs in up to 40% of patients and is due to the resulting prostaglandin-dependent vasodilatation (Hanson et al., 2012). Moreover, a recent study demonstrated that the pro-inflammatory effect of DMF administration on intestinal epithelial cells (IEC), which contributes to the gastrointestinal side effects possibly occurring in the earliest months of treatment, is also dependent on the experimental context (Parodi et al., 2021). In fact, the pathways activated through the binding of MMF to HCAR2 differed in *in vitro* and *in vivo* models, with the HCAR2/COX-2 pathway being responsible for the inflammation in IEC grown *in vitro*, and the HCAR2/extracellular signal-regulated kinases (ERK) 1/2 pathway being involved in the inflammatory response in IEC *in vivo*. Furthermore, the pro-inflammatory effects were only observed in resting, but not IFN-γ-stimulated IEC in the *in vitro* model, while the opposite was observed *in vivo*, where DMF activated the HCAR2/ERK1/2 pathway only in pro-inflammatory conditions (i.e., in EAE-affected, but not naïve, mice).

DMF also exhibits a regulatory effect on iron availability in specific cell types. Iron accumulation is another process that has been linked to the pathogenesis of neurodegenerative diseases. Physiologically, iron plays a key role in myelination, and is released by microglia in order to support the myelination process carried out by oligodendrocytes (Todorich et al., 2011). Damage to oligodendrocytes can cause uncontrolled iron release and extracellular accumulation, leading to an increase in free radicals and induction of the inflammatory response (Haider et al., 2014). DMF exhibits a neuroprotective effect against the iron-induced toxicity, modulating the microglial and oligodendrocytic levels of T-cell immunoglobulin mucin domain 2 protein (TIM-2) and transferrin receptor 1, which increase the uptake of iron and ferritin by these cells, shifting microglia back to an anti-inflammatory phenotype (Kronenberg et al., 2019).

## 3 Current approved clinical indications of DMF

### 3.1 Psoriasis

Psoriasis is a non-contagious dermatosis arising from a chronic inflammatory response, in which scaly skin plaques, easily distinguished from the surrounding normal epithelium, form as a result of the abnormal activity of different cell types, including macrophages, T lymphocytes, dendritic cells, neutrophils, and keratinocytes (Nograles and Krueger, 2011). The extension of the skin lesions, according to which the degree of severity is established, can vastly differ, from covering less than 2% of the skin surface (mild psoriasis), to affecting up to 10% (moderate psoriasis) or more (severe psoriasis) of the body (Lebwohl et al., 2004). As previously mentioned, in the late 1950s, when DMF was first examined as a therapeutic option for the treatment of psoriasis, the disease was thought to be the result of a metabolic dysfunction in the TCA cycle (Schweckendiek, 1959). It was hypothesized that FA, as an intermediate Krebs cycle product, could balance the enzymatic deficit, but esters of FA were administered instead, because of their favorable absorption profile (Altmeyer et al., 1994). Encouraged by the promising positive results obtained in clinical trials, a formulation containing DMF and salts of MEF, Fumaderm^®^, was approved for the treatment of moderate-to-severe plaque psoriasis (Altmeyer et al., 1994; Wollina, 2011). The sole administration of MEF did not exhibit any discernible clinical impact on the ailment, however, thus DMF was ascertained as the bioactive constituent of the medication. Another formulation containing exclusively DMF, Skilarence^®^, was approved shortly thereafter (Mrowietz et al., 2016). DMF is currently the first-line systemic therapy for chronic plaque psoriasis (Paolinelli et al., 2022), with the therapeutic dose of the drug ranging from 30 to 720 mg. A recent multicentric retrospective study on 103 patients affected by psoriasis and treated with DMF (Corazza et al., 2021) underlined an onset of efficacy consistent with previously available data, which indicated fumarates as comparatively slower than other therapies (de Jong et al., 1996). In fact, the Psoriasis Area and Severity Index (PASI) reached a 75% reduction (i.e., PASI75) after 12 weeks in 23% of patients (35% of those still undergoing DMF treatment), and after 26 weeks of continued treatment in 40% of patients (80% of patients who had not interrupted treatment), respectively (Corazza et al., 2021). The pathogenesis of psoriasis is still to be fully elucidated, but immunological factors play quite a significant role in its etiology (Ogawa et al., 2017), and thus DMF’s main mechanism of action in this pathological context was identified in its immunomodulatory properties (Montes Diaz et al., 2018). More specifically, the DMF-induced shift toward the Th2 phenotype in CD4^+^ cells contrasts the Th1 and Th17 lymphocytes infiltration of the skin, which triggers the dysfunction of keratinocytes and the skin lesions peculiar to psoriasis (Treumer et al., 2003). In addition, it is now clear that both the activation of the Nrf2 pathway and the inhibition of NF-kB activity regulate the pattern of cytokine release, promoting anti-inflammatory responses (Wilms et al., 2010; Ghoreschi et al., 2011).

### 3.2 Multiple sclerosis

DMF’s effectiveness in modulating the inflammatory processes responsible for plaque psoriasis promoted the exploration of FAEs therapeutic potential in mice with experimental autoimmune encephalomyelitis (EAE), a well-known *in vivo* model of MS (Schilling et al., 2006). Dysfunctions in the immune response, both innate and adaptive, are at the core of MS etiology, and lead to demyelinating lesions (Høglund, 2014). After a promising outcome in the EAE animal model, Fumaderm^®^ was administered for the first time to MS patients in 2006 (Schimrigk et al., 2006); the encouraging improvements observed in clinical and radiological parameters led to further clinical trials and eventually to the formulation of BG-12, a slow-release orally administered preparation of DMF. BG-12/DMF was confirmed to decrease both the ratio of relapsing patients, and the annual relapse rate and number of lesions per patient (Fox et al., 2012; Gold et al., 2012; Fox and Phillips, 2013). These effects are linked to the immunomodulatory activity of DMF, as suggested by the lower counts of both CD4^+^ and CD8^+^ T cells, dendritic cells (DCs) and B cells in treated patients (Longbrake et al., 2016). Moreover, DMF therapeutic effect also correlates with Nrf2 pathway activation (Hammer et al., 2018) and the modulation of the maturation process of DCs, and more specifically with their DMF-induced activation towards an anti-inflammatory phenotype (Ghoreschi et al., 2011; Peng et al., 2012). Despite the enteric coating of DMF pills, purposely formulated to reduce its GI tract-related side effects, GI symptoms are still very common in the first month of BG-12/DMF therapy and still constitute the main reason for the treatment discontinuation (Fox et al., 2016; Palte et al., 2019). This limitation motivated further efforts towards the improvement of DMF’s gut tolerability, resulting in the formulation of a novel FAE, diroximel fumarate (DRF). DRF has been marketed under the name Vumerity^®^ and has been approved for the treatment of RRMS after recent Phase III studies (NCT02634307, NCT03093324) that proved the safety and efficacy profiles of DRF (Palte et al., 2019; Naismith et al., 2020). Compared to DMF, DRF presents fewer GI-related adverse effects, and an equivalent production of MMF. The improvement in gut tolerability is likely linked to the different metabolites released following the esterase-dependent cleavage of DMF and DRF; in fact, DMF’s metabolism results in the release of MMF and methanol, the latter of which causes GI tract symptoms upon interaction with the small intestine mucosa. DRF’s main metabolites, on the other hand, are MMF and inert 2-hydroxyethyl succinimide, as well as lesser amounts of RDC-8439 and methanol (Palte et al., 2019).

## 4 Novel potential pharmacological applications

**FIGURE 2:**
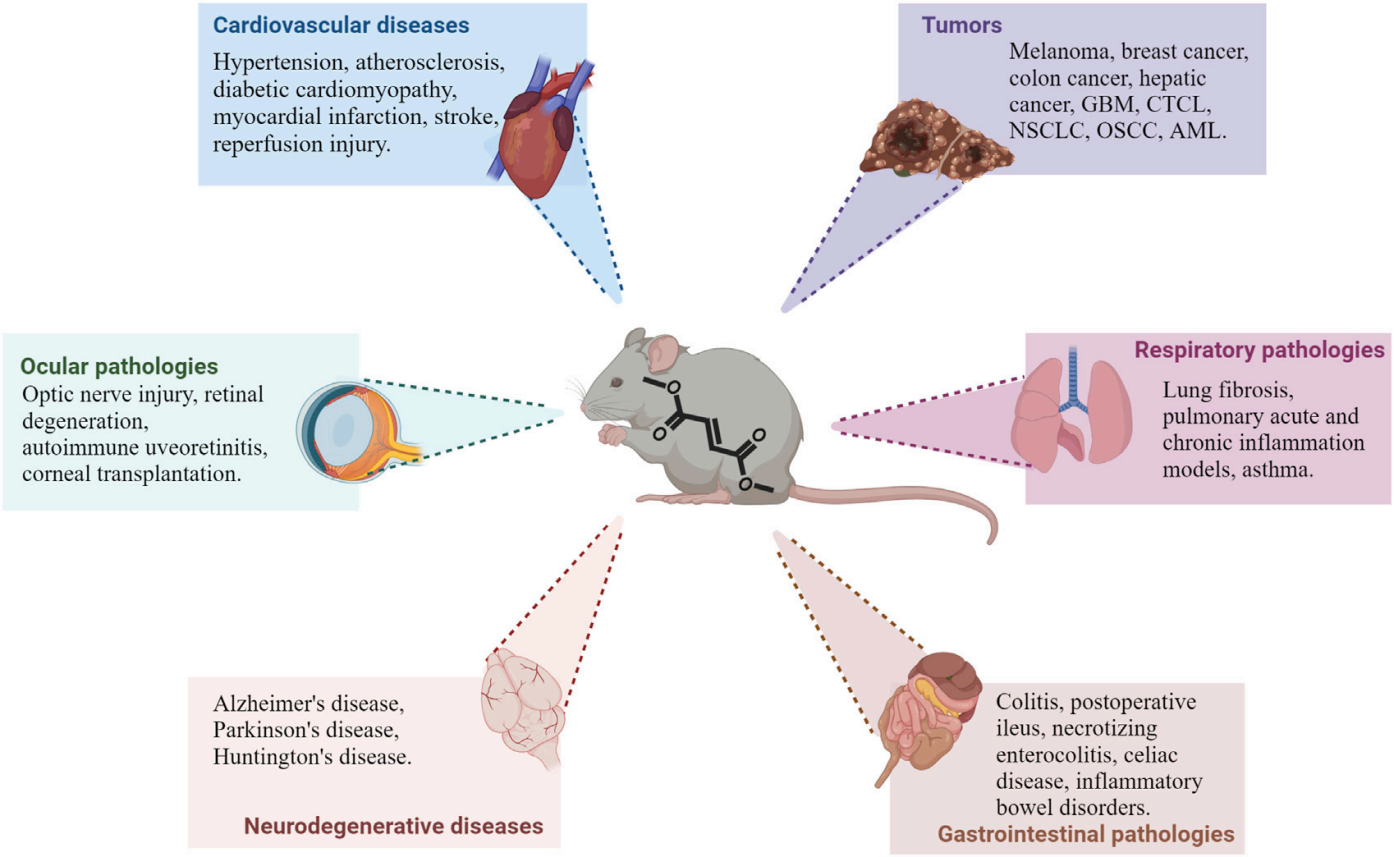
Main pathological contexts reporting protective or beneficial effects of DMF treatment. Recent studies demonstrate DMF’s efficacy in several *in vivo* models of different pathologies; in particular, preclinical data is available on different cardiovascular, neurodegenerative, ocular and gastrointestinal diseases, as well as tumors, as shown in the picture. DMF’s effect is also being investigated in other pathologies outside of these main contexts (see text for additional information). Abbreviations: GBM: glioblastoma multiforme; CTCL: cutaneous T-cell lymphoma; NSCLC: non-small cells lung cancer; OSCC: oral squamous cell carcinoma; AML: acute myeloid leukemia.

The immunomodulatory effects of DMF that have been discussed so far have contributed to entice interest in the potential repurposing of the compound in several new pathological contexts. Because of the molecular pathways involved in DMF’s therapeutic effects, its future applications will most likely focus on diseases in which inflammatory responses or oxidative stress are main factors in tiology (Figure 2). In the next sections, we discuss the latest findings on the effects of DMF and its potential for novel clinical applications (Table 1).

**TABLE 1:** Summary of human studies testing the effect of dimethyl fumarate for novel clinical applications.

Study (Author and year)	Study design	Study population	Treatment details (duration and dose)	Findings
Schmieder et al. (2015)	Prospective pilot study	Patients with psoriasis (n = 27)	16 weeks	• Increase in adiponectin
			600 mg/die	• Reduction of depressive symptoms
Blumenfeld Kan et al. (2019)	Retrospective study	Patients with multiple sclerosis (n = 41)	12 months dose N/A ⋮	• Increase in HDL, HDL/LDL ratio and HDL/total cholesterol
				• No change in LDL or triglycerides
Holzer et al. (2020)	Prospective, randomized controlled head-to-head trial	Patients with psoriasis (n = 65)	6 months	• Decrease in total cholesterol and apolipoprotein B
			120 mg/die	
Filippi et al. (2022)	Case report	Patient with psoriasis (n = 1)	9 weeks	• Improvements of working memory, writing, speech, and motor coordination
			30–720 mg/die	• Reduction in tremor and facial hypomimia
Vucic et al. (2021)	Phase-2, double-blind, placebo-controlled	Patients with amyotrophic lateral sclerosis (n = 107)	36 weeks	• Reduction in neurophysiological index
	randomized trial		480 mg/die	• No effect on respiratory function, urinary neurotrophin, and quality of life
				• Safe and well-tolerated
Kabanovski et al. (2023)	Prospective study	Patients with relapsing-remitting multiple	1–4 years dose N/A ⋮	• No effect on retinal nerve fiber layers
		sclerosis (n = 18)		
Heinz and Heiligenhaus (2007)	Pilot study	Patients with bilateral uveitis (n = 4)	12–15 months	• Improvement of cystoid macular edema and uveitis
			600–1,200 mg/die	
Kofler et al. (2018)	Case report	Patient with age-related macular degeneration (n = 1)	60 months	• Reduction in the retinal thickness
			240 mg/die	• Improvement of macular edema
Diebold et al. (2022)	Prospective study	Patients with relapsing-remitting multiple	12 months	• Alterations in the gut microbiota metabolome
		sclerosis (n = 20)	240 mg/twice die^a^	• Induction of lymphopenia
Eppinga et al. (2017)	Retrospective study	Patients with psoriasis (n = 90)	6–9 weeks dose N/A⁘ ⋮	• Induction of *Saccharomyces cerevisiae* growth
Katz Sand et al. (2019)	Cross-sectional study	Patients multiple sclerosis (n = 186)	At least 3 months dose N/A ⋮	• Decrease in the phyla *Firmicutes* and *Fusobacteria*
				• Increase in the phylum *Bacteroidetes*
Storm-Larsen et al. (2019)	Pilot trial	Patients with relapsing-remitting multiple	12 weeks dose N/A ⋮	• Normalization of the low abundance of butyrate-producing *Faecalibacterium*
		sclerosis (n = 36)		
Buscarinu et al. (2021)	Intervention trial	Patients with relapsing-remitting multiple	9 months dose N/A ⋮	• No clear effect on intestinal permeability
		sclerosis (n = 25)		• Reduction in the circulating levels of CD161+CCR6+CD8^+^ T cells
Milad et al. (2022)	Randomized, phase II, placebo, triple-blinded, controlled trial	Patients with glioblastoma (n = 36)	One week before surgery	• Higher Kanofsky’s performance status
			240 mg, three-times per day	• No change in the serum S100β level
Shafer et al. (2017)	Phase I single-arm	Patients with glioblastoma (n = 12)	DMF combined with radio- and chemotherapy	• Increase in progression-free survival
	single-institution		120 mg twice daily	• Safe and well tolerated
	dose-escalation study		240 mg twice and three times daily	

^a^ : Authors declare the study to be a continuation of a previous work (Diebold et al., 2018), where the specified treatment was 240 mg twice daily of (DMF, delayed release formulation).

^⋮^: Whenever not otherwise specified, the dose might be assumed as 240 mg/twice die, which is usually referred to as the recommended therapy (Storm Larsen et al., 2019; Phillips et al., 2015).

^⁘^: Authors defined the dosage of *in vitro* treatments as 1.6 mg/mL DMF, and 0.46 mg/mL fumaric acid, but did not state the dose administered to patients participating in the study.

### 4.1 Cardiovascular diseases

Several cardiovascular diseases, such as hypertension, atherosclerosis, diabetic cardiomyopathy, myocardial infarction, and stroke, are exacerbated by inflammation or oxidative stress (Bai et al., 2021; Tian et al., 2023). Studies on rat models of dexamethasone (DEX) and high-fat-diet-induced programmed hypertension (Hsu et al., 2019; Lin et al., 2018) revealed that 50 mg/kg/day of DMF induced upregulation of *Nrf2* mRNA expression, along with a decrease in asymmetric dimethylarginine (ADMA) plasma levels, and the downregulation of the renin–angiotensin system. The mechanisms through which the Nrf2 pathway can exert protective effects against inflammatory processes have been already discussed; reduced ADMA levels correlate with increased NO availability, which prevents DEX- and high-fat-diet-induced hypertension. A concomitant increase in mRNA levels of *UNC-51-like kinase-1* (*Ulk1*), *Ppargc1a*, and *autophagy-related gene 5* (*Atg5*) was also observed. An *in vivo* model of gestational hypertension in rats revealed that DMF oral treatment (25 mg/kg) decreases mRNA and protein levels of ten-eleven translocation 1 (TET1) and calcium-activated potassium channel subunit β1 (KCNMB1) (Zhou et al., 2021); TET1 is upregulated by estrogen during pregnancy and increases KCNMB1 level, which is associated with hypertension (Han et al., 2017).

DMF has been shown to decrease the severity of ischemic-derived neuronal damage in *vivo* mouse and rat models of middle cerebral artery occlusion (MCAO) and bilateral common carotid artery (BCCA) occlusion (Lin et al., 2016; Yao et al., 2016; Hatware and Akula, 2017; Safari et al., 2019; Hou et al., 2020; Owjfard et al., 2021). The dosage of DMF administered to the MCAO animal models varied through studies: 25 or 50 mg/kg twice daily (Lin et al., 2016), 30 or 45 mg/kg twice daily (Yao et al., 2016), 15 mg/kg twice daily (Safari et al., 2019), 12.5 mg/kg twice daily (Hou et al., 2020), 30 mg/kg daily (Owjfard et al., 2021). The outcomes of experiments testing various doses revealed that DMF’s protective properties are dose-dependent. The reduction of infarction volume, a DMF-induced rise in Nrf2 and HO-1 expression levels, a decrease in pro-inflammatory mediators like IL-1 and TNF, an increase in anti-inflammatory mediators like IL-10, and a suppression of T cell and neutrophil infiltration were all observed outcomes in the studies mentioned above. Nrf2 pathway activation was also identified as the main mechanism of action leading to the neuroprotective effects of DMF in rats and mice models of subarachnoid hemorrhage and intracerebral hemorrhage (Iniaghe et al., 2015; Liu et al., 2015; Zhao et al., 2015). Additionally, *in vivo* models of myocardial infarct demonstrated that 10 mg/kg of DMF reduced the infarct size by inhibiting NF-κB (Meili-Butz et al., 2008) and counteracting left ventricle wall thinning, concurrently improving collagen deposition, angiogenesis and myofibroblasts activation in the damaged area (Mouton et al., 2021). Reperfusion injury is a direct consequence of the interruption in blood flux. DMF has been tested in two *in vitro* models of reperfusion injury to determine how well it protects against damage caused by this condition because inflammation and oxidative stress are both involved (Kuang et al., 2020; Zhao et al., 2020). Both studies employed H9c2 cardiomyoblasts, subjected to oxygen-glucose deprivation/reoxygenation (OGD/R) and treated with 10 or 20 µM of DMF. They found that Nrf2 was upregulated, apoptotic cell death indicators were suppressed, ROS generation was reduced, and adhesion molecules were downregulated. More recent studies have observed the Nrf2-dependent protective and anti-inflammatory effect of DMF (15, 25, 45 or 100 mg/kg) on *in vivo* models of MCAO/reperfusion (Li et al., 2021b), myocardial ischemia-reperfusion injury in diabetic rats (Wang et al., 2023), and hepatic (Takasu et al., 2017; Ibrahim et al., 2020; Qi et al., 2023), intestinal (Gendy et al., 2021), cutaneous (Inoue et al., 2020) and renal (Ragab et al., 2020; Zhen et al., 2021) ischemia/reperfusion injury. Myocardial infarctions and ischemic strokes are both primarily caused by atherosclerosis because advanced atheromatous plaques are prone to rupture. *In vivo* models of diabetes or high-cholesterol-diet-induced atherosclerosis demonstrated that 25 mg/kg (Luo et al., 2019) and 12.5 mg/kg (Nour et al., 2017) of DMF induce the activation of the Nrf2 pathway, reduce aortic oxidation and serum TC, TGs, and LDL cholesterol. Moreover, a recent *in vitro* and *in vivo* study shows that 25 mg/kg of DMF inhibits the maturation and the migration of activated dendritic cells, which play an important role in atheromatous plaque rupture (Sun et al., 2022b). Diabetes can also result in cardiovascular symptoms; the impact of DMF on these consequences was examined in numerous recent research using *in vivo* models of streptozotocin-induced diabetes (Hu et al., 2018; Li et al., 2018; Amin et al., 2020; Lone et al., 2020), high-fat-diet-induced diabetes (Lu et al., 2017), or autoimmune diabetes (Li et al., 2021a). DMF treatment (with the administered dose ranging from 10 to 80 mg/kg) leads to a Nrf2-dependent protection against diabetic-induced myocardial injury and nephropathy, and to an improvement in wound healing. In the autoimmune model, DMF (25 mg/kg) delayed or prevented the onset of diabetes, and attenuated the levels of pro-inflammatory cytokines (Li et al., 2021a). Moreover, a streptozotocin- and high-fat-diet-induced diabetic rat model showed that DMF (25 mg/kg), administered alone or in combination with glibenclamide, alleviated fatty liver symptoms, usually associated with type II diabetes (Dwivedi and Jena, 2023).

An *in vivo* model of Vitamin D3-induced calcification underlined a preventive effect of DMF (25 or 50 mg/kg) against vascular calcification through the Nrf2-dependent regulation of genes linked to osteoblast-like phenotype and the reduction of calcium deposit (Ha et al., 2014). A recent study on an *in vivo* model of murine-elastase-induced aneurysm demonstrates the neuroprotective and preventive effects of 100 mg/kg DMF (Pascale et al., 2020). Lastly, DMF (25 mg/kg) has also been shown to reduce oxidative stress and inflammation in a rat model of isoproterenol-induced cardiac hypertrophy, resulting in a decrease in heart rate and blood pressure and heart-to-body weight ratio (Ahmed et al., 2018).

Notably, studies on patients affected by either plaque psoriasis or MS also report cardioprotective effects of DMF treatment (Schmieder et al., 2015; Blumenfeld Kan et al., 2019; Holzer et al., 2020). Patients affected by moderate-to-severe plaque psoriasis and treated with DMF showed a significant increase in serum levels of adiponectin (Schmieder et al., 2015) and decreases in apolipoprotein B and total cholesterol levels (Holzer et al., 2020). Blumenfeld Kan et al. observed a cohort of patients affected by multiple sclerosis and treated either with DMF or with fingolimod; the study reported increases in HDL level, HDL/LDL ratio and HDL/total cholesterol ratio, with no concurrent increase in LDL levels following both treatments.

### 4.2 Neurodegenerative diseases

Neurodegenerative diseases, e.g., Alzheimer’s disease, Parkinson’s disease, Huntington’s disease, amyotrophic lateral sclerosis, are characterized, among other features, by inflammation, oxidative stress, and the slow, progressive death of neurons (Guo et al., 2022; Teleanu et al., 2022). Thus, antioxidative and anti-inflammatory properties are sought after qualities in any therapeutic approach in these pathological contexts. Developing an animal model that reliably reflects the complexity of neurodegenerative diseases is still challenging, and several models aim to duplicate their main pathological features with different strategies. Studies on *in vivo* models of Alzheimer’s disease (AD) have demonstrated the efficacy of DMF therapy in reducing spatial memory and cognitive impairments, attenuating inflammation and oxidative stress and decreasing neurodegeneration. Majkutewicz et al. used an *in vivo rat* model of sporadic AD induced by intracerebroventricular injection of streptozotocin (STZ), and administered DMF through the chow (0.4% of food weight, corresponding to 44 mg/kg daily). DMF treatment led to protective effects against neurodegeneration in the hippocampus and basal forebrain and functional improvements in the Morris water maze test performance (Majkutewicz et al., 2016). The therapeutic effects exhibited by DMF were more significant in older rats compared to young animals (Majkutewicz et al., 2018). More recently, DMF was demonstrated to reduce the number of lymphocytes and the serum levels of IL-6 and IFN-γ (Wrona et al., 2022), and to improve neurogenesis and BDNF-related neuroprotection (Kurowska-Rucińska et al., 2022) in the same STZ-induced model. Transgenic animal models of amyloidosis were used to test neuroprotective properties of DMF, leading to conflicting results. In particular, a daily dose of 100 mg/kg of DMF reduces motor dysfunctions and improves cognitive function through the activation of the Nrf2 pathway in a transgenic mouse model of combined amyloidosis and tauopathy (Rojo et al., 2018), whereas no protective effect is observed after DMF treatment (75 mg/kg) in a mouse model combining overexpressed amyloid precursor protein and mutated presenilin-1 (Mohle et al., 2021). However, the difference between the two studies may be attributed to the timeline and length of treatment, which was not uniform among the two. Improvements in cognitive function and spatial memory, together with decreases in neurodegeneration of hippocampus and astrocyte activation, were also observed after DMF treatment (45 mg/kg) in a rat model of postmenopausal dementia (Abd El-Fatah et al., 2021). In another recent study using hippocampal injection of amyloid β and ibotenate acid to induce neurodegeneration, inflammation and memory impairment, a daily dose of DMF (48 mg/kg) reduced inflammation and oxidative stress in a Nrf2-dependent manner (Sun et al., 2022a). Recently, it has been suggested that transplantation of mesenchymal stem cells (MSCs) could represent a promising therapeutic option in neurodegenerative contexts; however, the potential of this treatment is hindered by the poor survival rate of MSCs after transplantation. A recent study demonstrated that preconditioning of MSCs cells with DMF leads to an increased survival rate and Nrf2 expression *in vitro*, and enhances MSCs efficacy in rescuing learning and spatial memory deficits in a rat model of amyloid β-induced AD (Babaei et al., 2023).

DMF has also been tested in *vivo* models of Parkinson’s disease (PD). Treatment with 50 mg/kg of DMF, administered prior to 6-hydroxy-dopamine (6-OHDA) lesioning, attenuated the loss of dopaminergic neurons, the decrease in dopamine levels and the activation of astrocytes and microglia (Jing et al., 2015). This mitigation of astrogliosis and microgliosis was also observed in a transgenic mouse model expressing human α-synuclein into the substantia nigra pars compacta, to which 100 mg/kg of DMF were administered daily for 1 and 3 weeks, and every other day for 8 weeks after vector delivery (Lastres-Becker et al., 2016). The study also reported a decrease in α-synuclein-mediated toxicity in dopaminergic neurons and an increase in intracellular levels of the protective factors NQO1 and oxidative stress induced growth inhibitor 1 (OSGIN1). Two studies describe DMF’s neuroprotective properties in a well-known mouse model of PD established through 1-methyl-4-phenyl-1,2,3,6-tetrahydropyridine (MPTP) injection (Ahuja et al., 2016; Campolo et al., 2017). In the former study, DMF treatments (10, 50, 100 mg/kg) started 1 day before MPTP administration and continued for 5 days after. Campolo et al. administered 10, 30, 100 mg/kg of DMF, starting 1 day after MPTP injection and continuing through 7 additional days after MPTP. DMF treatments prevent MPTP-induced loss of dopaminergic neurons and striatal dopamine, depletion of tyrosine hydroxylase, GSH and dopamine transporter, and reduce cellular levels of nitrative stress marker 3-nitrotyrosine (3-NT), TNF-α, monocyte chemoattractant protein-1 (MCP-1), α-synuclein oligomers, NF-κB and other oxidative stress or inflammatory markers. More recently, DMF (15–30–60 mg/kg) has been administered to a rotenone-induced mouse model of PD, leading to improvements in the rotenone-induced motor deficits and to decreases in the levels of α-synuclein in brain sections (Khot et al., 2023). Results from this study also remarked a DMF-induced activation of the autophagic pathway, and an inhibition of apoptosis and inflammatory cytokines.

Another study on DMF focused on the *DJ-1β* gene in *Drosophila*, considered to be an ortholog of human *DJ-1*. The DJ-1 gene is one of the genes involved in familial types of the PD and involved in both mitochondrial homeostasis and cellular defense against oxidative stress. DMF treatment partially reversed the motor deficits of this *in vivo* model, concurrently decreasing H_2_O_2_ and protein carbonylation levels and increasing phosphofructokinase activity (Solana-Manrique et al., 2020). In humans, a case report shows improvements in PD symptoms in a single patient affected by psoriasis and treated with DMF, administered daily starting from a dose of 30 mg and progressively increased to 720 mg at week 9 and beyond. The authors described improvements in working memory, writing, speech and motor coordination, combined with a reduction in tremor and facial hypomimia, likely due to DMF (Filippi et al., 2022).

Huntington’s disease (HD) research has also assessed the efficacy of DMF. R6/2 and YAC128 mice are two HD models, carrying a fragment of the human mHtt gene containing CAG repeats or the full gene, respectively; DMF counteracted motor impairment and increased the portion of preserved neurons in the striatum in both *in vivo* models, also leading to upregulation of Nrf2 immunoreactivity in neurons, an index of restored endogenous neuroprotection (Ellrichmann et al., 2011). In another study, rats were injected with 3-nitropropionic acid to induce selective progressive striatal degeneration. DMF (25 mg/kg) restored motor function, reduced neurodegeneration, and inhibited apoptosis (Hassab et al., 2023).

A Phase II randomised, placebo-controlled, double-blind clinical trial assessed the efficacy and safety of DMF in amyotrophic lateral sclerosis (ALS) patients (Vucic et al., 2021). Participants were administered either placebo or 480 mg or DMF daily, and efficacy measures were analysed at week 36. Specifically, changes in the ALS Functional Rating Scale-Revised, survival, quality of life, respiratory function, neurophysiological index (NI), and urinary neurotrophin-receptor p75 levels were evaluated. Except for a reduction in NI decline, DMF showed no significant effect in the other outcome measures, therefore the trial provided evidence for safety but lack of efficacy of DMF in ALS. However, the therapeutic potential of DMF in ALS may be better investigated on *in vivo* models of the disease, or through clinical trials on patients at different stages of the pathology, to account for potential differences in DMF’s efficacy on varying degrees of severity.

Clinical trials on DMF in any other mentioned neurodegenerative disease have not been yet carried out.

### 4.3 Respiratory pathologies

Complementary to the above-mentioned evidence in the context of cardiovascular diseases, DMF treatment exerts beneficial effects in experimental models of pulmonary arterial hypertension and lung fibrosis (Grzegorzewska et al., 2017). In particular, DMF (90 mg/kg) mitigated oxidative stress damage and inflammation in lungs of chronically hypoxic mice and showed protective effects in pulmonary arterial vascular smooth muscle cells and lung fibroblasts from human subjects. In agreement with this, *in vivo* studies confirmed the efficacy of DMF in an age-dependent persistent lung fibrosis mouse model (Kato et al., 2022). Lung-targeted (inhaled) delivery of 80 μg and systemic (oral) delivery of 240 μg of DMF, administered daily for 3–6 weeks post-injury, were compared in an aging murine model of bleomycin-induced non-resolving lung fibrosis; notably, only inhaled DMF restored lung Nrf2 levels, reduced lung oxidative stress, and promoted the fibrosis resolution. Relevant in the context of idiopathic pulmonary fibrosis, DMF-loaded ROS-responsive liposomes (containing DMF 0.5, 2, or 5 mg/kg) administered by inhalation attenuated lung fibrosis in animals through Nrf2 pathway upregulation (Liu C et al., 2022; Liu J et al., 2022); such a promising beneficial profile in a preclinical study clearly opens a new perspective for the DMF use in this clinical condition. Moreover, inhalation of 6 mg of DMF-encapsulated nanoparticles attenuated clinical signs of EAE and pulmonary inflammatory dysfunction in mice (Pinto et al., 2022).

Regarding a potential use of DMF in airways diseases, this drug has been anecdotally reported to reduce asthma symptoms in patients with psoriasis and asthma, and to provide beneficial effects on primary human lung cells (Seidel and Roth, 2013). In an experimental *in vivo* model of allergic asthma, intranasal administration of DMF (0.5 mg/kg) 30 min before the exposure to house dust led to a significant abrogation of the outcomes: DMF-treated animals showed reduced dust-induced airway inflammation, mucous cell metaplasia, and airway hyperactivity to inhaled methacholine (Jaiswal et al., 2019). Mechanistically, the anti-inflammatory effects of DMF were ascribed to interference with the migration of lung dendritic cells to mediastinal lymph nodes, and consequently to attenuation of allergic sensitization and Th2 immune response.

In the context of acute lung injury, in mice that underwent intratracheal instillation of lipopolysaccharide, a single dose of DMF (90 mg/kg by intrapetitoneal injection) ameliorated pulmonary injury and edema, and reduced infiltration of inflammatory mediators (Li et al., 2022a). In a mouse model of lung inflammation and oxidative stress induced by chronic exposure to diesel exhaust particles, animals exposed for 30 days to the toxic stimulus, and then to concomitant administration of DMF (30 mg/kg of DMF by gavage) for additional 30 days, showed reduced lung injury and inflammation, as well as lower oxidative stress, than their counterparts not treated with DMF (Cattani-Cavalieri et al., 2020).

In a mouse model of TNF-α-induced systemic inflammatory response syndrome, 50 mg/kg of DMF markedly suppressed the lesions of lung, as well as of cecum and uterus, concomitantly with a reduced production of pro-inflammatory cytokines (Shi et al., 2023); these DMF-protective effects were linked to inhibition of necroptosis triggered by TNF-α stimulation, suggesting for the first time that DMF may act as a necroptosis inhibitor.

Of interest for the recent pandemic coronavirus disease (COVID-19) and other viral infections, *in vitro* evidence showed that DMF 150–200 μM induces an interferon-independent antiviral program broadly effective in limiting virus replication and in suppressing the pro-inflammatory responses of various human pathogenic viruses, such as Herpes Simplex Virus-1 and-2, Zika virus, Vaccinia virus, Severe Acute Respiratory Syndrome Coronavirus 2 (Olagnier et al., 2020).

In virtue of its immunomodulatory and anti-inflammatory properties, the utility of DMF treatment in COVID-19 patients—especially those experiencing the cytokine storm—has been suggested (Timpani and Rybalka, 2020). A systematic review and network meta-analysis assessed the effect of disease-modifying therapies (DMTs) on COVID-19 gravity in MS patients, showing that DMF, as the majority of DMTs, was associated with a decreased risk of severe COVID-19 (Barzegar et al., 2022). Notably, a large data analysis from 12 data sources in 28 countries evaluating COVID-19 severity outcomes used DMF as a reference to compare other DMTs, confirming its favourable profile (Simpson-Yap et al., 2021). In agreement, a small descriptive study reported that 43/51 of MS patients taking DMTs including DMF experienced mild COVID-19, not requiring hospitalization (Al-Shammri et al., 2023); similarly, another study displayed that the same DMTs were not associated with an increased risk of severe COVID-19 (Smith et al., 2022). These evidences are encouraging; however, further retrospective studies in human subjects under DMF treatment and infected by COVID-19 would be useful to determine the factual capability of DMF in modulating the host immune response to SARS-CoV-2 infection and preventing excessive inflammation and tissue injury associated with COVID-19 pathology in the airway and beyond.

### 4.4 Gastrointestinal pathologies

The gastro-enteric tract plays a central role in the maintenance of health homeostasis, for both its obvious part in nutrient absorption and its residing microbiota, which is closely connected to immunological as well as metabolic functions (Adak and Khan, 2019). Moreover, several studies suggest the involvement of the gut microbiome in the development of a number of pathologies, e.g., MS (Preiningerova et al., 2022), psoriasis (Buhas et al., 2022).

Recent evidence suggests a potential connection between the adverse effects of DMF treatment and its effects on gut microbiota. Changes in the composition of gut microbiota under DMF treatment have been indeed proposed as potential risk factors for lymphopenia (Diebold et al., 2022) and GI tract symptoms (Storm-Larsen et al., 2019). On the other hand, both preclinical studies on *in vivo* models and clinical studies on psoriasis patients have demonstrated some benefic properties exerted by DMF on the composition of the microbiota (Eppinga et al., 2017; Ma et al., 2017), suggesting a more complex interaction between the two.

Ma et al. administered DMF to BALB/c mice (0.05% of the feed administered to the animals) and observed remarkable improvements in the gut barrier, in growth performance and mucosal morphology, combined with increases in the diversity of the microbiota and its healthier composition. This led the Authors to conclude that DMF positively modulates absorption of nutrients and microbiota richness.

Eppinga et al. reported that DMF treatment restored *Saccharomyces cerevisiae* levels in a cohort of patients affected by plaque psoriasis; however, the study also underlines a positive correlation between higher amounts of *S. cerevisiae* and GI tract symptoms, suggesting that its higher abundance might lead to less favourable effects.

On MS patients, several clinical studies on subjects undergoing DMF treatment analysed the composition of the gut microbiome (Katz Sand et al., 2019; Storm-Larsen et al., 2019; Diebold et al., 2022; Ferri et al., 2023), observing changes in the relative abundance of different subpopulations. Some of the detected DMF-induced alterations, e.g., decreases in *Clostridium* (Ferri et al., 2023) and proinflammatory *taxa* like *A. muciniphilia* and *C. eutactus* levels and increases in anti-inflammatory species like *L. pentosus* (Diebold et al., 2022), potentially suggest that a positive modulation of the gut microbiome could represent an additional therapeutic mechanism of DMF.

DMF has also been shown to improve other aspects of the health of the GI tract. A study on the intestinal permeability (IP) and circulating CD161^+^CCR6^+^CD8^+^T cells in 25 MS patients underlined a significant decrease in the observed lymphocytic population after 9 months of DMF treatment (Buscarinu et al., 2021). The Authors also registered a significant correlation between circulating CD161^+^CCR6^+^CD8^+^T cells, IP changes and the clinical MRI parameters considered in the study, concluding that a modulation of this subset of T-cells could then correlate with disease course and therapy.

Evidence on the protective properties of DMF on the gastroenteric system also comes from *in vivo* models of colitis; DMF (10, 30, and 100 mg/kg) administered to a murine model of dinitrobenzene sulphuric acid (DNBS)-induced colitis reduced weight loss, hemorrhagic diarrhoea, colon injury, and colonic TNF-α and IL-1β levels (Casili et al., 2016). DMF also reduced NF-κB p65 nuclear expression, DNBS-induced lipid peroxidation through the regulation of Mn-superoxide dismutase, myeloperoxidase (MPO) activity and intracellular adhesion molecule (ICAM-) 1 levels, which contributes to cell recruitment during the inflammatory process. Two studies confirmed the same antioxidant and anti-inflammatory properties of DMF on dextran sulfate sodium (DSS)-induced colitis (Liu et al., 2016; Li et al., 2020). In the first case, 30 and 60 mg/kg of DMF were administered to mice after exposure to DSS for 7 days. DMF led to a dose-dependent attenuation of DSS-induced body weight decrease, colon shortening and splenomegaly, to the suppression of MPO and inducible nitric oxide synthase activity, to the reduction of inflammatory cell infiltration, of pro-inflammatory cytokine production and NLPR3 inflammasome activation. DMF also restored Nrf2 protein level and *NQO1* and *H O -1* expression; Li et al. (2020) further investigated in the DSS-induced colitis model the DMF’s effect on the gut antioxidant system, with specific focus on glutamate-cysteine ligase catalytic subunit (GCLC), glutathione peroxidase (GPX) and COX-2. The administration of 25 mg/kg of DMF led to a significant increase in GCLC and GPX expression, and to a reduction in the expression of inflammatory mediator COX-2.

In a murine model of postoperative ileus, induced via intestinal manipulation (IM), animals were administered DMF intraperitoneally (30 mg/kg) or intragastrically (100 mg/kg) 24 h prior to IM. DMF pretreatment prevented the impairment of GI motility which characterizes postoperative ileus, reduced IL-6 levels and leukocyte infiltration, and reduced NF-κB and ERK 1/2 activation (Van Dingenen et al., 2019).

Intestinal damage was also reduced in an *in vivo* model of necrotizing enterocolitis (NEC), in which 25 mg/kg of DMF attenuated the weight loss, abdominal distension diarrhoea and intestinal injuries derived from NEC (Mi et al., 2023). In the same animals, at intestinal level DMF also inhibited the expression of pro-inflammatory and pro-apoptotic factors.

The potential repurposing of DMF in the context of chronic inflammatory diseases of the intestine, such as inflammatory bowel disorders and celiac disease, has been extensively discussed in a recent review reporting that DMF actually overturned from main suspect to potential ally against gut disorders (Manai et al., 2022).

### 4.5 Ocular pathologies

The use of DMF has a strong attractiveness also in the ophthalmological context, where it may be beneficial in more than one eye disease (Manai et al., 2022). The rationale of positioning DMF in ocular pathologies indeed relies on evidence related to several diseases. First, studies of an animal model of MS and patients with RRMS show that systemic administration of DMF mitigates optic neuritis (Zyla et al., 2019), a demyelinating symptom affecting up to 50% of MS patients who experience monocular vision loss. Evidence-based improvements in optic neuritis, associated with preservation of both peripapillary retinal nerve fiber layer and ganglion cell layer in animal model of MS as well as in MS patients treated with DMF, have been also reported (Zyla et al., 2019; You et al., 2021). The beneficial effect on the neural retina in MS seems not to be exclusive of DMF, since a recent prospective study of retinal atrophy in RRMS patients treated with various disease-modifying therapies including DMF shows no difference among treatments (Kabanovski et al., 2023). However, as the Authors declare, limitations of their cohort study include both the high rate of patients drop out (41 of 109, 37.6%) and the slightly higher proportion of patients with history of optic neuritis (over 70%) and shorter treatment duration in the DMF group, which may have introduced a selection bias.

Two *in vivo* studies of optic nerve injury (Mori et al., 2020) and light-induced retinal degeneration (Dietrich et al., 2021) further highlight pro-survival effects of DMF on different cellular layers within the retina. In particular, the former study reports that DMF administration (100 mg/kg) protected retinal ganglion cells by degeneration, while the latter study shows a significant reduction in photoreceptors loss after DMF treatment (15 or 30 mg/kg). Coherently, intraperitoneal injection of MMF (100 mg/kg), the main DMF metabolite, exerted neuroprotective effects in a light-induced retinopathy animal model (Jiang et al., 2019).

*In vivo* evidence indicates that DMF treatment (100 mg/kg) had a beneficial effect in an experimental autoimmune uveoretinitis rat model and may represent a potential candidate drug for ocular inflammatory diseases (Labsi et al., 2021). In addition, one pilot study in human subjects showed robust, positive effects of DMF/Fumaderm^®^ administration (600–1,200 mg/die for 12–15 months) in counteracting the symptoms of uveitis and cystoid macular edema (Heinz and Heiligenhaus, 2007). Another case report of cystoid macular edema, probably in the context of age-related macular degeneration (AMD), demonstrated that DMF/Fumaderm^®^ (240 mg/day) improves retinal thickness and other symptoms in an old woman followed up for 60 months (Kofler et al., 2018).

An *in vivo* streptozotocin-induced diabetic rat model demonstrated the efficacy of DMF (10 mg/kg) in decreasing the retinal expression levels of key enzymes involved in diabetic retinopathy (DR). In particular, DMF administration resulted in a reduction in iNOS and COX-2 retinal levels and an induction of the expression of HO-1, suggesting an attenuation of inflammatory processes and oxidative response in DR (Giunta et al., 2023).

Concerning AMD, a randomized, open labelled phase II trial (NCT04292080, still recruiting on April the 19^th^, 2023) started in 2022, with the aim to evaluate the safety and efficacy of DMF/Tecfidera^®^ in two groups of patients (treated vs. untreated) with geographic atrophy, the late stage of dry AMD. Specifically, to slow the growth of areas of geographic atrophy, patients will be treated twice a day with 120 mg for the first week, and then 240 mg for 51 weeks.

Of interest, *in vivo* literature evidence of beneficial effects exerted by MMF, either orally or intraocularly administered, is consistent with the DMF-induced improvement of retinal health (for a comprehensive review, see Manai et al., 2022).

Subconjunctival injections of DMF (20 µg) promoted corneal allograft survival in rats undergoing corneal transplantation by inhibiting lymphangiogenesis and macrophage infiltration responsible for transplant rejection (Yu et al., 2021). Moreover, treatment with DMF (15 mg/kg) for 28 days pre-infection and 14 days post-infection with *Herpes simplex* Virus 1 (HSV1) improved severe keratitis by reducing corneal inflammation in mice (Heiligenhaus et al., 2004). The antiviral effect of DMF against HSV1 has been recently confirmed *in vitro* by a study also investigating the topical use of DMF loaded in ethosome gel, which displays favourable skin tolerability and safety in human volunteers (Sicurella et al., 2023). Of interest, recent *in vitro* evidence in human primary retinal endothelial cells showed cytoprotective and antioxidant effects of DMF under high glucose conditions, opening an interesting perspective and rationale for further evaluation of this molecule in retinal pathologies featured by vascular complications, such as diabetic retinopathy (Manai et al., 2022).

The studies of DMF effectiveness by local application in ocular pathologies, although still limited in number and disease contexts, encourage putting further efforts in the development of novel topical formulations and delivery systems for the eye. Compared to oral treatment, DMF ocular administration by injection or, preferably, eye drops/gel, indeed might allow to have various advantages, such as avoidance or reduction of both systemic side effects and targeting to other organs, and possibility to treat also patients presenting ADME alterations (Manai et al., 2022).

### 4.6 Tumors

DMF’s antitumoral properties are being studied in several types of cancer. DMF anti-proliferative effect against tumoral cells is mainly linked to its regulation of the nuclear translocation of NF-kB, of the Nrf2 pathway, of extracellular signal-regulated kinase 1 and 2 (ERK1/2), and p38 mitogen-activated protein kinases (MAPKs), while its suppression of tumor metastasis is connected to inhibition of matrix metalloproteinases (MMPs) and very late antigens (VLAs) (Saidu et al., 2019; Takeda et al., 2020). Some of the earliest evidence of DMF’s potential in inhibiting tumor growth came from its application to *in vitro* and *in vivo* models of melanoma (Loewe et al., 2006). DMF (6 mg/kg or 20 mg/kg) administered daily to a mouse model of melanoma significantly reduced tumor growth, mean tumor volume and tumor metastasis. These results were confirmed and expanded in successive studies, in which DMF was administered alone (Takeda et al., 2020) or in combination with other therapies (Valero et al., 2010; Li et al., 2022a). In particular, Takeda et al., 2020, administered 10 or 30 mg/kg of DMF to a mouse model of melanoma, observing a DMF-induced decrease in tumor growth and increase in survival time. Valero et al., 2010, analyzed the effects of 20 mg/kg of DMF, alone or in combination with alkylating agent dacarbazine; both treatments reduced tumor growth, and their combination delayed metastasis and impaired melanoma cell migration. Li et al., 2022b, investigated the effect of DMF (6 mg/kg) and vemurafenib co-treatment; in agreement with the previously mentioned studies, the Authors showed that tumor growth was significantly impaired by DMF administration, and that DMF enhanced the efficacy of vemurafenib in the group that received the combined treatment.

Early evidence of DMF’s anti-tumoral activity was also found in *vivo* studies on azoxymethane (AOM)-induced foci of aberrant crypts in rat colon (Pereira et al., 1994; Rao et al., 1995). AOM-induced foci are precancerous lesions used as biomarkers for bioassays of chemopreventive agents; among various screened compounds, Pereira et al., 1994, found that DMF (400 or 800 mg/kg) reduced the yield of AOM-induced foci of aberrant crypts. Rao et al., 1995, also assayed DMF’s efficacy in reducing AOM-induced foci in rat colon, observing a significant inhibition of invasive adenocarcinoma incidence and multiplicity. More recently, DMF (2 mg/kg) was shown to potentiate the antitumor activity of mitomycin C in a mouse model of colon cancer (Begleiter et al., 2004; Saidu et al., 2017, assayed DMF on both *in vitro* and *in vivo* models; DMF (>25 μM) proved to have a cytotoxic effect in several cancer lines. Moreover, administration of DMF 20 mg/kg to two mice models of colon cancer, provided further *in vivo* evidence of its efficacy in decreasing tumor occurrence and growth, mainly by dampening tumor-promoting chronic inflammation (Saidu et al., 2017).

Two studies investigate the potential therapeutic value of DMF in the treatment of breast cancer. Digby et al., 2005, analyzed DMF’s efficacy as an anti-inflammatory adjuvant option, administering it through the diet as 0.3% of the food weight to enhance the effect of the novel bioreductive agent RH1 (2,5-diaziridinyl-3-(hydroxymethyl)-6-methyl-1,4-benzoquinone). The Authors did not remark on a significant antitumoral activity of RH1, but they did notice a DMF-induced increase in NQO1, suggesting a beneficial effect on the antioxidative system (Digby et al., 2005; Kastrati et al., 2016, reported for the first time the therapeutic efficacy of 30 mg/kg of DMF in both *in vitro* and *in vivo* models of breast cancer, observing impaired tumor growth after DMF treatment (Kastrati et al., 2016).

A phase II randomized, triple-blind controlled trial on 36 patients affected by glioblastoma multiforme (GBM) investigated the effect of DMF administration (240 mg three times per day) prior to surgery, followed by standard treatments (radiotherapy and chemotherapy with temozolomide). The study observed serum S100β levels and Kanofsky’s performance status (KPS) score, indexes of brain injury and the ability of patients to carry out daily tasks, respectively; the Authors remark no significant change in serum S100β level, but a higher mean KPS score, suggesting that DMF’s neuroprotective action might not be due to the prevention of BBB integrity disruption, but to other mechanisms (Milad et al., 2022). A phase I clinical trial on GBM patients administered DMF along the standard therapies, providing further evidence for the safety of DMF in combination with radio- and chemotherapy in this pathological context (Shafer et al., 2017).

Other types of tumor, which DMF likely has beneficial effects against, include cutaneous T-cell lymphoma (CTCL) (Nicolay et al., 2016; Nicolay et al., 2023), non-small cell lung cancer (NSCLC) (Rupp et al., 2022), hepatic cancer (Dwivedi and Jena, 2020), acute myeloid leukemia (AML) (Nachliely et al., 2019), oral squamous cell carcinoma (OSCC) (Basilotta et al., 2023).

Nicolay et al., 2016, investigated the efficacy of 30 mg/kg of DMF in two *in vivo* models of CTCL with different cutaneous localizations of the T-cell infiltrate; DMF proved to be effective in delaying tumor growth, preventing metastasis and inducing cell death in primary tumors and liver metastases. Results from this preclinical study were then translated in a multicentric phase II study (NCT02546440). DMF (starting from 30 mg/die, escalating weekly by 30 mg/die up to 120 mg/die, then by 120 mg/d up to a final dose of 720 mg/die) was administered to 25 patients with CTCL over a period of 24 weeks; efficacy (assessed through skin involvement, blood involvement, pruritus and quality of life) and safety were then evaluated. In 30.4% of patients a significant (>50%) reduction of skin involvement (measured with the Modified Severity-Weighted Assessment Tool) was registered, with better responses shown by patients with high tumor burden in skin and blood. Pruritus also decreased in several patients, and data from blood samples confirmed DMF-induced NF-κB-inhibition. Authors concluded DMF to be an effective therapeutic option for CTCL, with excellent tolerability (Nicolay et al., 2023).

Rupp et al., 2022, administered 30 mg/kg of DMF to a mouse model of NSCLC, successfully repressing tumor growth and reducing tumor cell proliferation.

Dwivedi and Jena, 2020, used a two-stage model of early hepatic carcinogenesis, established through the administration of thioacetamide (TAA) and diethylnitrosamine (DEN), to test the efficacy of DMF in hepatocarcinogenesis. DMF treatment (25 mg/kg) resulted in improvements in all DEN + TAA-induced alterations (e.g., decrease in body weight, increase in liver weight, histopathological alterations, apoptosis insensitivity, DNA damage, and dysfunctions in the antioxidant, inflammatory, fibrogenic and regenerative proliferative stress pathways).

In a xenograft mouse model of AML, Nachliely et al., 2019, combined DMF (0.6 mg) with vitamin D derivatives (VDDs); when administered alone, DMF and the VDD reduced tumor size and weight, but the reduction is much more significant when the compounds are administered in combination.

Basilotta et al., 2023, administered 30 or 100 mg/kg of DMF to an orthotopic mouse model of OSCC; the mass of the tumors, as was the extent of the neutrophilic infiltration, were reduced in a dose-dependent manner. Basilotta and others also observed a significantly higher expression levels of caspase 3, BCL2-associated agonist of cell death, HO-1 and manganese superoxide dismutase in samples obtained from the OSCC mouse model.

Among several observational studies investigating a potential correlation between DMF and cancer, two pharmacovigilance cohort studies evaluated MS patients who were being treated with disease-modifying therapies (DMTs) and who also reported cases of cancer; the two consulted databases were WHO’s VigiBase^®^ (Dolladille et al., 2021) and FDA’s Adverse Event Reporting System (Stamatellos et al., 2021), respectively. The first study analysed 2,40,993 reports related to DMTs prescribed to MS patients, of which DMF made up 10% of cases; the Authors underline a significant association between cancer reporting and DMF treatment, as well as other DMTs like natalizumab, interferon-β and fingolimod. On the other hand, Stamatellos and others considered data from 2004 to 2020, including in their analysis all reports of patients undergoing any of the FDA-approved treatments for MS, but removing cases of patients younger than 9 years old, and receiving concomitant treatment with antineoplastic drugs. None of the DMTs was significantly associated with increased risk of reporting cancer (Stamatellos et al., 2021). Notably, none of DMF-containing drugs’ package inserts and reports indicate “tumors” as contraindications and adverse effects up to now.

### 4.7 Other pathologies

The beneficial effects exerted by DMF make it easy to conceive its repurposing in other pathologies whose pathogeneses rely on inflammation and/or an altered immune response. A paradigmatic example is given by systemic lupus erythematosus (SLE) and similar manifestations. This autoimmune disorder (i.e., type III hypersensitivity response) is characterized by the loss of homeostasis of the immune system, which mistakenly attacks healthy tissue leading to the onset of several symptoms, such as painful joint, fever, ulcers, swollen lymph nodes and skin rash (Ceccarelli et al., 2023). Generally, SLE is associated with cardiovascular comorbidities, which represent the first cause of death (Lisnevskaia et al., 2014). Different studies demonstrated that DMF is able to reduce plasmablast differentiation and antibody production in SLE (Mauro et al., 2023) as well as T-cell metabolism and functionality (Kell et al., 2023). Both studies employed samples obtained from SLE patients, from which cells were isolated and treated with DMF 25 μM. Notably, encouraging results were obtained also for discoid lupus erythematosus (Fumaderm^®^ administered orally 1 up to 3 tables per day for 6 months) (Tsianakas et al., 2014) and cutaneous lupus erythematosus (CLE) (Fumaderm^®^, initially at 20 mg of DMF +75 mg MEF and then increased to 120 mg of DMF +95 mg of MEF), as a result of a phase II clinical trial (NCT01352988) (Kuhn et al., 2016). Interestingly, as highlighted by Tsianakas and others (2014), different studies and case reports demonstrated the efficacy of DMF in SLE treatment compared with cases in which canonical therapies were ineffective.

DMF may be also useful as adjuvant therapy in sepsis. A strong preventing antioxidant activity of DMF has indeed been observed in a rat model of organ sepsis; 15 mg/kg of DMF administered immediately and 12 h after sepsis induction resulted in reduced neutrophil infiltration in peripheral organs (i.e., liver, lung), and in the prevention of nitrite/nitrate increase and oxidative damage to lipids (measured through the production of MDA equivalents) and proteins (measured through carbonyl content), and restored superoxide dismutase (SOD) and catalase activity (Giustina et al., 2017).

The neuroprotective properties of DMF seem to be beneficial also in some hereditary metabolic diseases, as in the case of the X-linked adrenomyeloneuropathy (AMN). This disorder, which is characterized by a plethora of symptoms, also presents spinal cord dysfunction and peripheral neuropathy. When Abcd1-mice, an animal model of adrenomyeloneuropathy, were fed chow containing 100 mg/kg of DMF, it had been found that the Nrf2 pathway was rescued, the antioxidant system was improved, and inflammation in the spinal cord was prevented (Ranea-Robles et al., 2018). In addition, DMF-fed Abcd1^−^/Abcd2^−/−^ mice, a model of X-linked adrenoleukodystrophy (X-ALD), showed reversal of astrocytosis, microgliosis, axonal and myelin degeneration in their spinal cord along with an improvement in locomotor function (Ranea-Robles et al., 2018). In various models of Friedreich’s ataxia (FRDA) (reviewed by Seminotti et al., 2021), DMF, which acts on Nrf2 signaling that appears impaired in this condition, demonstrated promising effects.

Furthermore, other genetic disorders that affect the peripheral nervous system could benefit from DMF treatment, such as Friedrich’s ataxia (reviewed by Seminotti et al., 2021). Particularly, orally administration of DMF (110 mg/kg/day) was able to rescue mitochondrial gene expression in brain and to restore frataxin expression in brain, cerebellum, and quadriceps muscle in a mouse model of Friedrich’s ataxia (Hui et al., 2021). These results were corroborated by another study based on animal models and human patients. Similar to the previous study, Friedrich’s ataxia subjects were treated using the approved protocol of 240 mg twice daily for 3 months (Jasoliya et al., 2019). The Nrf2-dependent mechanism of action of DMF is particularly intriguing in the context in Friedreich’s ataxia since the only drug recently approved by FDA for the treatment of these patients works by acting on the Nrf2 pathway (Dayalan Naidu and Dinkova-Kostova, 2023).

## 5 Conclusion

The present work highlights the potential of DMF as stand-alone or adjuvant therapy in several disorders beyond psoriasis and RRMS. Considering its critical role in the modulation of various signaling networks associated with multiple physiological and pathological processes (e.g., oxidative damage, inflammation, and autophagy), DMF appears to be a versatile molecule with a wide spectrum of possible therapeutic applications. Although the anticancer mechanism of action remains elusive, several reports indicate that DMF may be an effective inhibitor of cancer cell growth, and thus potentially useful in breast and lung cancers, glioblastomas, and T-cell lymphoma. In addition, due to its involvement in diverse biological activities, including vascular protective effects, DMF has the potential to be repurposed as a therapeutic agent for patients with cardiovascular diseases. Likewise, with its combination of antioxidant and anti-inflammatory effects, DMF represents a promising neuroprotective molecule in the management of neurodegenerative diseases. Other recent evidence indicates that DMF has effects at ocular level, opening a new perspective for its repositioning in eye pathologies characterized by oxidative stress and inflammation. Studies on the gut microbiota also demonstrate a rearrangement of specific *taxa* upon DMF treatment with a decrease of proinflammatory *taxa* and an increase in beneficial anti-inflammatory species; moreover, literature on DMF and gut disorders intriguingly demonstrates its potential applications in inflammatory and immune-mediated intestinal diseases.

The evidence collected in this review highlights the efficacy of DMF-based approaches in different pathological contexts and strongly demonstrates that this molecule deserves more attention by the scientific community. Indeed, the majority of the summarized findings is drawn from preclinical studies and represents the starting point for future in depth research aimed at strengthening these evidences and transfer them from the bench to the clinic.

## References

[B1] Abd El-Fatah I. M. Abdelrazek H. M. A. Ibrahim S. M. Abdallah D. M. El-Abhar H. S. (2021). Dimethyl fumarate abridged tauo-/amyloidopathy in a D-Galactose/ovariectomy-induced Alzheimer’s-like disease: Modulation of AMPK/SIRT-1, AKT/CREB/BDNF, AKT/GSK-3β, adiponectin/Adipo1R, and NF-κB/IL-1β/ROS trajectories. Neurochem. Int. 148, 105082. 10.1016/j.neuint.2021.105082 34052296

[B2] Adak A. Khan M. R. (2019). An insight into gut microbiota and its functionalities. Cell Mol. Life Sci. 76, 473–493. 10.1007/s00018-018-2943-4 30317530 PMC11105460

[B3] Ahmed A. A. Ahmed A. A. E. El Morsy E. M. Nofal S. (2018). Dimethyl fumarate interferes with MyD88-dependent toll-like receptor signalling pathway in isoproterenol-induced cardiac hypertrophy model. J. Pharm. Pharmacol. 70, 1521–1530. 10.1111/jphp.13000 30175489

[B4] Ahuja M. Ammal Kaidery N. Yang L. Calingasan N. Smirnova N. Gaisin A. (2016). Distinct Nrf2 signaling mechanisms of fumaric acid esters and their role in neuroprotection against 1-methyl-4-phenyl-1,2,3,6-tetrahydropyridine-induced experimental Parkinson’s-like disease. J. Neurosci. 36, 6332–6351. 10.1523/JNEUROSCI.0426-16.2016 27277809 PMC4899530

[B5] Al-Ani M. Elemam N. M. Hundt J. E. Maghazachi A. A. (2020). Drugs for multiple sclerosis activate natural killer cells: Do they protect against COVID-19 infection? Infect. Drug Resist 13, 3243–3254. 10.2147/IDR.S269797 33061471 PMC7519863

[B6] Al-Shammri S. Chadha G. Chattopadhyay A. Doi S. (2023). The impact of SARS-CoV-2 infection in multiple sclerosis patients taking disease- modifying therapies. Med. Princ. Pract. 1. 10.1159/000530764 PMC10319087 37231972

[B7] Altmeyer P. J. Mattlies U. Pawlak F. Hoffmann K. Frosch P. J. Ruppert P. (1994). Antipsoriatic effect of fumaric acid derivatives. Results of a multicenter double-blind study in 100 patients. J. Am. Acad. Dermatol 30, 977–981. 10.1016/s0190-9622(94)70121-0 8188891

[B8] Amin F. M. Abdelaziz R. R. Hamed M. F. Nader M. A. Shehatou G. S. G. (2020). Dimethyl fumarate ameliorates diabetes-associated vascular complications through ROS-TXNIP-NLRP3 inflammasome pathway. Life Sci. 256, 117887. 10.1016/j.lfs.2020.117887 32497629

[B9] Aubets J. Jansat J. Salva M. Birks V. M. Cole R. J. Lewis J. (2019). No evidence for interactions of dimethylfumarate (DMF) and its main metabolite monomethylfumarate (MMF) with human cytochrome P450 (CYP) enzymes and the P‐glycoprotein (P‐gp) drug transporter. Pharmacol. Res. Perspect. 7, e00540. 10.1002/prp2.540 31832203 PMC6887663

[B10] Azzi A. (2022). Oxidative stress: What is it? Can it Be measured? Where is it located? Can it Be good or bad? Can it Be prevented? Can it Be cured? Antioxidants 11, 1431. 10.3390/antiox11081431 35892633 PMC9329886

[B11] Babaei H. Kheirollah A. Ranjbaran M. Cheraghzadeh M. Sarkaki A. Adelipour M. (2023). Preconditioning adipose-derived mesenchymal stem cells with dimethyl fumarate promotes their therapeutic efficacy in the brain tissues of rats with Alzheimer's disease. Biochem. Biophys. Res. Commun. 672, 120–127. 10.1016/j.bbrc.2023.06.045 37348174

[B12] Bai R. Lang Y. Shao J. Deng Y. Refuhati R. Cui L. (2021). The role of NLRP3 inflammasome in cerebrovascular diseases pathology and possible therapeutic targets. ASN Neuro 13, 17590914211018100. 10.1177/17590914211018100 34053242 PMC8168029

[B13] Barzegar M. Houshi S. Sadeghi E. Hashemi M. S. Pishgahi G. Bagherieh S. (2022). Association of disease-modifying therapies with COVID-19 susceptibility and severity in patients with multiple sclerosis: A systematic review and network meta-analysis. Mult. Scler. Int. 2022, 9388813. 10.1155/2022/9388813 36187599 PMC9519336

[B14] Basilotta R. Lanza M. Filippone A. Casili G. Mannino D. De Gaetano F. (2023). Therapeutic potential of dimethyl fumarate in counteract oral squamous cell carcinoma progression by modulating apoptosis, oxidative stress and epithelial–mesenchymal transition. Int. J. Mol. Sci. 24, 2777. 10.3390/ijms24032777 36769105 PMC9917022

[B15] Begleiter A. Leith M. K. Thliveris J. A. Digby T. (2004). Dietary induction of NQO1 increases the antitumour activity of mitomycin C in human colon tumours *in vivo* . Br. J. Cancer 91, 1624–1631. 10.1038/sj.bjc.6602171 15467770 PMC2409939

[B16] Blumenfeld Kan S. Staun-Ram E. Golan D. Miller A. (2019). HDL-cholesterol elevation associated with fingolimod and dimethyl fumarate therapies in multiple sclerosis. Mult. Scler. J. Exp. Transl. Clin. 5, 2055217319882720. 10.1177/2055217319882720 31662882 PMC6794658

[B17] Buhas M. C. Gavrilas L. I. Candrea R. Catinean A. Mocan A. Miere D. (2022). Gut microbiota in psoriasis. Nutrients 14, 2970. 10.3390/nu14142970 35889927 PMC9321451

[B18] Buscarinu M. C. Gargano F. Lionetto L. Capi M. Morena E. Fornasiero A. (2021). Intestinal permeability and circulating CD161+CCR6+CD8+T cells in patients with relapsing–remitting multiple sclerosis treated with dimethylfumarate. Front. Neurol. 12, 683398. 10.3389/fneur.2021.683398 34512507 PMC8426620

[B19] Campolo M. Casili G. Biundo F. Crupi R. Cordaro M. Cuzzocrea S. (2017). The neuroprotective effect of dimethyl fumarate in an MPTP-mouse model of Parkinson’s disease: Involvement of reactive oxygen species/nuclear factor-κb/nuclear transcription factor related to NF-E2. Antioxid. Redox Signal 27, 453–471. 10.1089/ars.2016.6800 28006954 PMC5564046

[B20] Casili G. Cordaro M. Impellizzeri D. Bruschetta G. Paterniti I. Cuzzocrea S. (2016). Dimethyl fumarate reduces inflammatory responses in experimental colitis. J. Crohns Colitis 10, 472–483. 10.1093/ecco-jcc/jjv231 26690241 PMC4946754

[B21] Cattani-Cavalieri I. da Maia Valença H. Moraes J. A. Brito-Gitirana L. Romana-Souza B. Schmidt M. (2020). Dimethyl fumarate attenuates lung inflammation and oxidative stress induced by chronic exposure to diesel exhaust particles in mice. Int. J. Mol. Sci. 21, 9658. 10.3390/ijms21249658 33352854 PMC7767202

[B22] Ceccarelli F. Perricone C. Natalucci F. Picciariello L. Olivieri G. Cafaro G. (2023). Organ damage in systemic lupus erythematosus patients: A multifactorial phenomenon. Autoimmun. Rev. 22, 103374. 10.1016/j.autrev.2023.103374 37301273

[B23] Chen H. Assmann J. C. Krenz A. Rahman M. Grimm M. Karsten C. M. (2014). Hydroxycarboxylic acid receptor 2 mediates dimethyl fumarate’s protective effect in EAE. J. Clin. Invest 124, 2188–2192. 10.1172/JCI72151 24691444 PMC4001545

[B25] Corazza M. Odorici G. Conti A. Di Lernia V. Motolese A. Bardazzi F. (2021). Dimethyl fumarate treatment for psoriasis in a real‐life setting: A multicentric retrospective study. Dermatol Ther. 34, e15066. 10.1111/dth.15066 34291547 PMC9286462

[B26] Cuadrado A. Kügler S. Lastres-Becker I. (2018). Pharmacological targeting of GSK-3 and NRF2 provides neuroprotection in a preclinical model of tauopathy. Redox Biol. 14, 522–534. 10.1016/j.redox.2017.10.010 29121589 PMC5681345

[B27] Dayalan Naidu S. Dinkova-Kostova A. T. (2023). Omaveloxolone (Skyclarys^TM^) for patients with Friedreich's ataxia. Trends Pharmacol. Sci. 44, 394–395. 10.1016/j.tips.2023.03.005 37142519

[B28] de Jong R. Bezemer A. C. Zomerdijk T. P. L. van de Pouw-Kraan T. Ottenhoff T. H. M. Nibbering P. H. (1996). Selective stimulation of T helper 2 cytokine responses by the anti-psoriasis agent monomethylfumarate. Eur. J. Immunol. 26, 2067–2074. 10.1002/eji.1830260916 8814248

[B29] Diebold M. Meola M. Purushothaman S. Siewert L. K. Poessnecker E. Roloff T. (2022). Gut microbiota composition as a candidate risk factor for dimethyl fumarate-induced lymphopenia in multiple sclerosis. Gut Microbes 14, 2147055. 10.1080/19490976.2022.2147055 36398902 PMC9677991

[B30] Dietrich M. R. Hecker C. Nasiri M. Samsam S. Issberner A. Kohne Z. (2021). Neuroprotective properties of dimethyl fumarate measured by optical coherence tomography in non-inflammatory animal models. Front. Neurol. 11, 601628. 10.3389/fneur.2020.601628 33519681 PMC7838501

[B31] Digby T. Leith M. K. Thliveris J. A. Begleiter A. (2005). Effect of NQO1 induction on the antitumor activity of RH1 in human tumors *in vitro* and *in vivo* . Cancer Chemother. Pharmacol. 56, 307–316. 10.1007/s00280-004-0961-4 15877230

[B32] Dolladille C. Chrétien B. Peyro-Saint-Paul L. Alexandre J. Dejardin O. Fedrizzi S. (2021). Association between disease-modifying therapies prescribed to persons with multiple sclerosis and cancer: A WHO pharmacovigilance database analysis. Neurotherapeutics 18, 1657–1664. 10.1007/s13311-021-01073-y 34231126 PMC8608969

[B33] Duran A. Amanchy R. Linares J. F. Joshi J. Abu-Baker S. Porollo A. (2011). p62 is a key regulator of nutrient sensing in the mTORC1 pathway. Mol. Cell 44, 134–146. 10.1016/j.molcel.2011.06.038 21981924 PMC3190169

[B34] Dwivedi D. K. Jena G. (2020). Diethylnitrosamine and thioacetamide-induced hepatic damage and early carcinogenesis in rats: Role of Nrf2 activator dimethyl fumarate and NLRP3 inhibitor glibenclamide. Biochem. Biophys. Res. Commun. 522, 381–387. 10.1016/j.bbrc.2019.11.100 31761320

[B35] Dwivedi D. K. Jena G. (2023). Dimethyl fumarate‐mediated Nrf2/ARE pathway activation and glibenclamide‐mediated NLRP3 inflammasome cascade inhibition alleviate type II diabetes‐associated fatty liver in rats by mitigating oxidative stress and inflammation. J. Biochem. Mol. Toxicol. 37, e23357. 10.1002/jbt.23357 36999408

[B36] Ellrichmann G. Petrasch-Parwez E. Lee D-H. Reick C. Arning L. Saft C. (2011). Efficacy of fumaric acid esters in the R6/2 and YAC128 models of Huntington’s disease. PLoS ONE 6, e16172. 10.1371/journal.pone.0016172 21297955 PMC3031519

[B37] Eppinga H. Thio H. B. Marco W. Schreurs J. Blakaj B. Tahitu R. I. (2017). Depletion of *Saccharomyces cerevisiae* in psoriasis patients, restored by Dimethylfumarate therapy (DMF). PLoS ONE 12, e0176955. 10.1371/journal.pone.0176955 28486503 PMC5423625

[B38] Ferri C. Castellazzi M. Merli N. Laudisi M. Baldin E. Baldi E. (2023). Gut microbiota changes during dimethyl fumarate treatment in patients with multiple sclerosis. Int. J. Mol. Sci. 24, 2720. 10.3390/ijms24032720 36769041 PMC9917003

[B39] Filippi F. Zengarini C. Sacchelli L. Bardazzi F. Conti A. Lasagni C. (2022). Use of Apremilast® in the psoriasis treatment: A real-life multicenter Italian experience. Ital. J. Dermatol Venerol. 157, 313–317. 10.23736/S2784-8671.21.07125-5 34545728

[B40] Fox E. J. Vasquez A. Grainger W. Ma T. S. von Hehn C. Walsh J. (2016). Gastrointestinal tolerability of delayed-release dimethyl fumarate in a multicenter, open-label study of patients with relapsing forms of multiple sclerosis (MANAGE). Int. J. MS Care 18, 9–18. 10.7224/1537-2073.2014-101 26917993 PMC4766951

[B41] Fox R. Phillips J. (2013). BG-12 in multiple sclerosis. Semin. Neurol. 33, 056–065. 10.1055/s-0033-1343796 23709213

[B42] Fox R. J. Miller D. H. Phillips J. T. Hutchinson M. Havrdova E. Kita M. (2012). Placebo-controlled phase 3 study of oral BG-12 or glatiramer in multiple sclerosis. N. Engl. J. Med. 367, 1087–1097. 10.1056/NEJMoa1206328 22992072

[B43] Gendy A. Soubh A. Al-Mokaddem A. Kotb El-Sayed M. (2021). Dimethyl fumarate protects against intestinal ischemia/reperfusion lesion: Participation of Nrf2/HO-1, GSK-3β and Wnt/β-catenin pathway. Biomed. Pharmacother. 134, 111130. 10.1016/j.biopha.2020.111130 33348309

[B44] Ghoreschi K. Brück J. Kellerer C. Deng C. Peng H. Rothfuss O. (2011). Fumarates improve psoriasis and multiple sclerosis by inducing type II dendritic cells. J. Exp. Med. 208, 2291–2303. 10.1084/jem.20100977 21987655 PMC3201195

[B45] Gillard G. O. Collette B. Anderson J. Chao J. Scannevin R. H. Huss D. J. (2015). DMF, but not other fumarates, inhibits NF-κB activity *in vitro* in an Nrf2-independent manner. J. Neuroimmunol. 283, 74–85. 10.1016/j.jneuroim.2015.04.006 26004161

[B46] Giunta S. D’Amico A. G. Maugeri G. Bucolo C. Romano G. L. Rossi S. (2023). Drug-repurposing strategy for dimethyl fumarate. Pharmaceuticals 16, 974. 10.3390/ph16070974 37513886 PMC10386358

[B47] Giustina A. D. Bonfante S. Zarbato G. F. Danielski L. G. Mathias K. de Oliveira A. N. (2017). Dimethyl fumarate modulates oxidative stress and inflammation in organs after sepsis in rats. Inflammation 41, 315–327. 10.1007/s10753-017-0689-z 29124567

[B48] Gold R. Kappos L. Arnold D. L. Bar-Or A. Giovannoni G. Selmaj K. (2012). Placebo-controlled phase 3 study of oral BG-12 for relapsing multiple sclerosis. N. Engl. J. Med. 367, 1098–1107. 10.1056/NEJMoa1114287 22992073

[B49] Grzegorzewska A. P. Seta F. Han R. Czajka C. A. Makino K. Stawski L. (2017). Dimethyl Fumarate ameliorates pulmonary arterial hypertension and lung fibrosis by targeting multiple pathways. Sci. Rep. 7, 41605. 10.1038/srep41605 28150703 PMC5288696

[B50] Guo S. Wang H. Yin Y. (2022). Microglia polarization from M1 to M2 in neurodegenerative diseases. Front. Aging Neurosci. 14, 815347. 10.3389/fnagi.2022.815347 35250543 PMC8888930

[B51] Ha C-M. Park S. Choi Y-K. Jeong J-Y. Oh C. J. Bae K-H. (2014). Activation of Nrf2 by dimethyl fumarate improves vascular calcification. Vasc. Pharmacol. 63, 29–36. 10.1016/j.vph.2014.06.007 25135648

[B52] Haider L. Simeonidou C. Steinberger G. Hametner S. Grigoriadis N. Deretzi G. (2014). Multiple sclerosis deep grey matter: The relation between demyelination, neurodegeneration, inflammation and iron. J. Neurol. Neurosurg. Psychiatry 85, 1386–1395. 10.1136/jnnp-2014-307712 24899728 PMC4251183

[B53] Hammer A. Waschbisch A. Kuhbandner K. Bayas A. Lee D. Duscha A. (2018). The NRF2 pathway as potential biomarker for dimethyl fumarate treatment in multiple sclerosis. Ann. Clin. Transl. Neurol. 5, 668–676. 10.1002/acn3.553 29928650 PMC5989754

[B54] Han Y. Y. Wang L. J. Zhang L. Zhang W. W. Ma K. T. Li L. (2017). Association between potassium channel SNPs and essential hypertension in Xinjiang Kazak Chinese patients. Exp. Ther. Med. 14, 1999–2006. 10.3892/etm.2017.4734 28962116 PMC5609208

[B55] Hanson J. Gille A. Offermanns S. (2012). Role of HCA₂ (GPR109A) in nicotinic acid and fumaric acid ester-induced effects on the skin. Pharmacol. Ther. 136, 1–7. 10.1016/j.pharmthera.2012.06.003 22743741

[B56] Hassab L. Y. Abbas S. S. Mohammed R. A. Abdallah D. M. (2023). Dimethyl fumarate abrogates striatal endoplasmic reticulum stress in experimentally induced late-stage Huntington’s disease: Focus on the IRE1α/JNK and PERK/CHOP trajectories. Front. Pharmacol. 14, 1133863. 10.3389/fphar.2023.1133863 37056990 PMC10088517

[B57] Hatware K. V. Akula A. (2017). Evaluation of safety profile and cerebroprotective potential of dimethyl fumarate (DMF) against ischemia and reperfusion induced cerebral injury in wistar rats. Int. J. Pharm. Pharm. Sci. 9, 241. 10.22159/ijpps.2017v9i2.16098

[B58] Hayden M. S. Ghosh S. (2008). Shared principles in NF-kappaB signaling. Cell 132, 344–362. 10.1016/j.cell.2008.01.020 18267068

[B59] Hayes J. D. Dinkova-Kostova A. T. (2014). The Nrf2 regulatory network provides an interface between redox and intermediary metabolism. Trends Biochem. Sci. 39, 199–218. 10.1016/j.tibs.2014.02.002 24647116

[B60] Heiligenhaus A. Li H. Wasmuth S. Bauer D. (2004). Influence of dimethyl fumarate on experimental HSV-1 necrotizing keratitis. Graefes Arch. Clin. Exp. Ophthalmol. 242 (10), 870–877. 10.1007/s00417-004-0933-8 15241613

[B61] Heinz C. Heiligenhaus A. (2007). Improvement of noninfectious uveitis with fumaric acid esters: Results of a pilot study. Arch. Ophthalmol. 125, 569–571. 10.1001/archopht.125.4.569 17420383

[B62] Hoffmann C. Dietrich M. Herrmann A-K. Schacht T. Albrecht P. Methner A. (2017). Dimethyl fumarate induces glutathione recycling by upregulation of glutathione reductase. Oxid. Med. Cell Longev. 2017, 6093903–6093908. 10.1155/2017/6093903 28116039 PMC5237454

[B63] Høglund R. A. Maghazachi A. A. (2014). Multiple sclerosis and the role of immune cells. World J. Exp. Med. 4, 27–37. 10.5493/wjem.v4.i3.27 25254187 PMC4172701

[B64] Holzer G. Hoke M. Sabeti‐Sandor S. Perkmann T. Rauscher A. Strassegger B. (2020). Disparate effects of adalimumab and fumaric acid esters on cardiovascular risk factors in psoriasis patients: Results from a prospective, randomized, observer‐blinded head‐to‐head trial. J. Eur. Acad. Dermatol Venereol. 35, 441–449. 10.1111/jdv.16635 32426884

[B65] Hou X. Xu H. Chen W. Zhang N. Zhao Z. Fang X. (2020). Neuroprotective effect of dimethyl fumarate on cognitive impairment induced by ischemic stroke. Ann. Transl. Med. 8, 375. 10.21037/atm.2020.02.10 32355819 PMC7186746

[B66] Hsu C-N. Lin Y-J. Yu H-R. LinChun I. Sheen J-M. Huang L-T. (2019). Protection of male rat offspring against hypertension programmed by prenatal dexamethasone administration and postnatal high-fat diet with the Nrf2 activator dimethyl fumarate during pregnancy. Int. J. Mol. Sci. 20, 3957. 10.3390/ijms20163957 31416234 PMC6719242

[B67] Hu X. Rajesh M. Zhang J. Zhou S. Wang S. Sun J. (2018). Protection by dimethyl fumarate against diabetic cardiomyopathy in type 1 diabetic mice likely via activation of nuclear factor erythroid-2 related factor 2. Toxicol. Lett. 287, 131–141. 10.1016/j.toxlet.2018.01.020 29408448

[B68] Hu Y. Luo Y. Zheng Y. (2022). Nrf2 pathway and autophagy crosstalk: New insights into therapeutic strategies for ischemic cerebral vascular diseases. Antioxidants 11, 1747. 10.3390/antiox11091747 36139821 PMC9495910

[B69] Hui C. K. Dedkova E. N. Montgomery C. Cortopassi G. (2021). Dimethyl fumarate dose-dependently increases mitochondrial gene expression and function in muscle and brain of Friedreich’s ataxia model mice. Hum. Mol. Genet. 29, 3954–3965. 10.1093/hmg/ddaa282 33432356 PMC8485216

[B70] Ibrahim S. G. El-Emam S. Z. Mohamed E. A. Abd Ellah M. F. (2020). Dimethyl fumarate and curcumin attenuate hepatic ischemia/reperfusion injury via Nrf2/HO-1 activation and anti-inflammatory properties. Int. Immunopharmacol. 80, 106131. 10.1016/j.intimp.2019.106131 31981960

[B71] Ichimura Y. Waguri S. Sou Y. S. Kageyama S. Hasegawa J. Ishimura R. (2013). Phosphorylation of p62 activates the Keap1-Nrf2 pathway during selective autophagy. Mol. Cell 51, 618–631. 10.1016/j.molcel.2013.08.003 24011591

[B72] Inami Y. Waguri S. Sakamoto A. Kouno T. Nakada K. Hino O. (2011). Persistent activation of Nrf2 through p62 in hepatocellular carcinoma cells. J. Cell Biol. 193, 275–284. 10.1083/jcb.201102031 21482715 PMC3080263

[B73] Iniaghe L. O. Krafft P. R. Klebe D. W. Omogbai E. K. I. Zhang J. H. Tang J. (2015). Dimethyl fumarate confers neuroprotection by casein kinase 2 phosphorylation of Nrf2 in murine intracerebral hemorrhage. Neurobiol. Dis. 82, 349–358. 10.1016/j.nbd.2015.07.001 26176793 PMC4640980

[B74] Inoue Y. Uchiyama A. Sekiguchi A. Yamazaki S. Fujiwara C. Yokoyama Y. (2020). Protective effect of dimethyl fumarate for the development of pressure ulcers after cutaneous ischemia‐reperfusion injury. Wound Repair Regen. 28, 600–608. 10.1111/wrr.12824 32356363

[B75] Jain A. Lamark T. Sjøttem E. Larsen K. B. Awuh J. A. Øvervatn A. (2010). p62/SQSTM1 is a target gene for transcription factor NRF2 and creates a positive feedback loop by inducing antioxidant response element-driven gene transcription. J. Biol. Chem. 285, 22576–22591. 10.1074/jbc.M110.118976 20452972 PMC2903417

[B76] Jaiswal A. K. Sandey M. Suryawanshi A. Cattley R. C. Mishra A. (2019). Dimethyl fumarate abrogates dust mite-induced allergic asthma by altering dendritic cell function. Immun. Inflamm. Dis. 7, 201–213. 10.1002/iid3.262 31264384 PMC6688084

[B77] Jasoliya M. Sacca F. Sahdeo S. Chedin F. Pane C. Brescia Morra V. (2019). Dimethyl fumarate dosing in humans increases frataxin expression: A potential therapy for Friedreich’s ataxia. PLoS ONE 14, e0217776. 10.1371/journal.pone.0217776 31158268 PMC6546270

[B78] Jiang D. Y. Ryals R. C. Huang S. H. Weller K. K. Titus H. Robb B. M. (2019). Monomethyl fumarate protects the retina from light-induced retinopathy. Invest Ophthalmol. Vis. Sci. 60, 1275–1285. 10.1167/iovs.18-24398 30924852 PMC6440526

[B79] Jing X. Shi H. Zhang C. Ren M. Han M. Wei X. (2015). Dimethyl fumarate attenuates 6-OHDA-induced neurotoxicity in SH-SY5Y cells and in animal model of Parkinson’s disease by enhancing Nrf2 activity. Neuroscience 286, 131–140. 10.1016/j.neuroscience.2014.11.047 25449120

[B80] Kabanovski A. Zaslavsky K. Rotstein D. Margolin E. (2023). A prospective study of disease modifying therapy and retinal atrophy in relapsing-remitting multiple sclerosis. J. Neurol. Sci. 446, 120552. 10.1016/j.jns.2023.120552 36774748

[B81] Kappos L. Gold R. Miller D. H. MacManus D. G. Havrdova E. Limmroth V. (2008). Efficacy and safety of oral fumarate in patients with relapsing-remitting multiple sclerosis: A multicentre, randomised, double-blind, placebo-controlled phase IIb study. Lancet 372, 1463–1472. 10.1016/S0140-6736(08)61619-0 18970976

[B82] Kastrati I. Siklos M. I. Calderon-Gierszal E. L. El-Shennawy L. Georgieva G. Thayer E. N. (2016). Dimethyl fumarate inhibits the nuclear factor κB pathway in breast cancer cells by covalent modification of p65 protein. J. Biol. Chem. 291, 3639–3647. 10.1074/jbc.M115.679704 26683377 PMC4751401

[B83] Kato K. Papageorgiou I. Shin Y. J. Kleinhenz J. M. Palumbo S. Hahn S. (2022). Lung-targeted delivery of dimethyl fumarate promotes the reversal of age-dependent established lung fibrosis. Antioxidants (Basel) 11, 492. 10.3390/antiox11030492 35326142 PMC8944574

[B84] Katz Sand I. Zhu Y. Ntranos A. Clemente J. Cekanaviciute E. Brandstadter R. (2019). Disease-modifying therapies alter gut microbial composition in MS. Neurol. Neuroimmunol. Neuroinflamm 6, e517. 10.1212/NXI.0000000000000517 30568995 PMC6278850

[B85] Kell L. Taylor S. Shah K. De Maeyer R. Isenberg D. Castelino M. (2023). Dimethyl fumarate modulates t-cell metabolism and function in systemic lupus erythematosus patient samples. Ann. Rheum. Dis. 82, 1241–1. 10.1136/annrheumdis-2023-eular.5460

[B86] Khot M. Sood A. Kamatham P. T. Pinjala P. Srivastava S. Singh S. B. (2023). Dimethyl fumarate ameliorates Parkinsonian pathology by modulating autophagy and apoptosis via Nrf2-TIGAR-LAMP2/Cathepsin D axis. Brain Res. 1815, 148462. 10.1016/j.brainres.2023.148462 37315723

[B87] Kobayashi A. Kang M-I . Okawa H. Ohtsuji M. Zenke Y. Chiba T. (2004). Oxidative stress sensor Keap1 functions as an adaptor for cul3-based E3 ligase to regulate proteasomal degradation of Nrf2. Mol. Cell Biol. 24, 7130–7139. 10.1128/MCB.24.16.7130-7139.2004 15282312 PMC479737

[B88] Kofler L. Kathrein-Schneider S. Schweinzer K. Kofler H. (2018). Fumaric acid: A possible new therapy for macular edema? Int. Ophthalmol. 39, 1627–1631. 10.1007/s10792-018-0982-3 29959659

[B89] Kourakis S. Timpani C. A. de Haan J. B. Gueven N. Fischer D. Rybalka E. (2020). Dimethyl fumarate and its esters: A drug with broad clinical utility? Pharmaceuticals 13, 306. 10.3390/ph13100306 33066228 PMC7602023

[B90] Kronenberg J. Pars K. Brieskorn M. Prajeeth C. K. Heckers S. Schwenkenbecher P. (2019). Fumaric acids directly influence gene expression of neuroprotective factors in rodent microglia. Int. J. Mol. Sci. 20, 325. 10.3390/ijms20020325 30650518 PMC6358967

[B91] Kuang Y. Zhang Y. Xiao Z. Xu L. Wang P. Ma Q. (2020). Protective effect of dimethyl fumarate on oxidative damage and signaling in cardiomyocytes. Mol. Med. Rep. 22, 2783–2790. 10.3892/mmr.2020.11342 32945364 PMC7453509

[B92] Kuhn A. Landmann A. Patsinakidis N. Ruland V. Nozinic S. Perusquía Ortiz A. M. (2016). Fumaric acid ester treatment in cutaneous lupus erythematosus (CLE): A prospective, open-label, phase II pilot study. Lupus 25, 1357–1364. 10.1177/0961203316644335 27147621

[B93] Kurowska-Rucińska E. Ruciński J. Myślińska D. Grembecka B. Wrona D. Majkutewicz I. (2022). Dimethyl fumarate alleviates adult neurogenesis disruption in Hippocampus and olfactory bulb and spatial cognitive deficits induced by intracerebroventricular streptozotocin injection in young and aged rats. Int. J. Mol. Sci. 23, 15449. 10.3390/ijms232415449 36555093 PMC9779626

[B94] Labsi M. Soufli I. Belguendouz H. Djebbara S. Hannachi L. Amir Z-C. (2021). Beneficial effect of dimethyl fumarate on experimental autoimmune uveitis is dependent of pro-inflammatory markers immunomodulation. Inflammopharmacology 29, 1389–1398. 10.1007/s10787-021-00864-1 34518966

[B95] Landeck L. Asadullah K. Amasuno A. Pau-Charles I. Mrowietz U. (2018). Dimethyl fumarate (DMF) vs. monoethyl fumarate (MEF) salts for the treatment of plaque psoriasis: A review of clinical data. Arch. Dermatol Res. 310, 475–483. 10.1007/s00403-018-1825-9 29574575 PMC6060759

[B96] Lastres-Becker I. García-Yagüe A. J. Scannevin R. H. Casarejos M. J. Kügler S. Rábano A. (2016). Repurposing the NRF2 activator dimethyl fumarate as therapy against synucleinopathy in Parkinson’s disease. Antioxid. Redox Signal 25, 61–77. 10.1089/ars.2015.6549 27009601 PMC4943471

[B97] Lebwohl M. Menter A. Koo J. Feldman S. R. (2004). Combination therapy to treat moderate to severe psoriasis. J. Am. Acad. Dermatol 50, 416–430. 10.1016/j.jaad.2002.12.002 14988684

[B98] Li H. Li M. Dong C. Liu B. (2022b). Dimethyl fumarate ameliorates lipopolysaccharide-induced acute lung injury by inhibiting NLRP3 inflammasome-mediated pyroptosis through enhancing Nrf2 signaling. Toxicol. Res. 11, 437–450. 10.1093/toxres/tfac020 PMC9244226 35782648

[B99] Li H. Wang Y. Su R. Jia Y. Lai X. Su H. (2022a). Dimethyl fumarate combined with vemurafenib enhances anti-melanoma efficacy via inhibiting the hippo/YAP, NRF2-ARE, and AKT/mTOR/ERK pathways in A375 melanoma cells. Front. Oncol. 12, 794216. 10.3389/fonc.2022.794216 35141161 PMC8820202

[B100] Li S. Takasu C. Lau H. Robles L. Vo K. Farzaneh T. (2020). Dimethyl fumarate alleviates dextran sulfate sodium-induced colitis, through the activation of nrf2-mediated antioxidant and anti-inflammatory pathways. Antioxidants 9, 354. 10.3390/antiox9040354 32344663 PMC7222424

[B101] Li S. Vaziri N. D. Swentek L. Takasu C. Vo K. Stamos M. J. (2021a). Prevention of autoimmune diabetes in NOD mice by dimethyl fumarate. Antioxidants 10, 193. 10.3390/antiox10020193 33572792 PMC7912218

[B102] Li Y. Chu L. Liu C-F. Zha Z. Shu Y. (2021b). Protective effect of GSK-3β/nrf2 mediated by dimethyl fumarate in middle cerebral artery embolization reperfusion rat model. Curr. Neurovasc Res. 18, 456–464. 10.2174/1567202618666211109105024 34751118

[B103] Li Y. Ma F. Li H. Song Y. Zhang H. Jiang Z. (2018). Dimethyl fumarate accelerates wound healing under diabetic condition. J. Mol. Endocrinol. 61, 163–172. 10.1530/JME-18-0102 30038053

[B104] Lin R. Cai J. Kostuk E. W. Rosenwasser R. Iacovitti L. (2016). Fumarate modulates the immune/inflammatory response and rescues nerve cells and neurological function after stroke in rats. J. Neuroinflammation 13, 269. 10.1186/s12974-016-0733-1 27733178 PMC5062839

[B105] Lin Y-J. Lin I-C. Yu H-R. Sheen J-M. Huang L-T. Tain Y-L. (2018). Early postweaning treatment with dimethyl fumarate prevents prenatal dexamethasone- and postnatal high-fat diet-induced programmed hypertension in male rat offspring. Oxid. Med. Cell Longev. 2018, 5343462–5343468. 10.1155/2018/5343462 29636848 PMC5832129

[B106] Linker R. A. Haghikia A. (2016). Dimethyl fumarate in multiple sclerosis: Latest developments, evidence and place in therapy. Ther. Adv. Chronic Dis. 7, 198–207. 10.1177/2040622316653307 27433310 PMC4935836

[B107] Linker R. A. Lee D-H. Ryan S. van Dam A. M. Conrad R. Bista P. (2011). Fumaric acid esters exert neuroprotective effects in neuroinflammation via activation of the Nrf2 antioxidant pathway. Brain 134, 678–692. 10.1093/brain/awq386 21354971

[B108] Lisnevskaia L. Murphy G. Isenberg D. (2014). Systemic lupus erythematosus. Lancet 384, 1878–1888. 10.1016/S0140-6736(14)60128-8 24881804

[B109] Litjens N. H. R. Burggraaf J. van Strijen E. van Gulpen C. Mattie H. Schoemaker R. C. (2004). Pharmacokinetics of oral fumarates in healthy subjects. Br. J. Clin. Pharmacol. 58, 429–432. 10.1111/j.1365-2125.2004.02145.x 15373936 PMC1884599

[B110] Liu C. Wang Y. Li Y. Tang J. Hong S. Hong L. (2022). Time-dependent dual beneficial modulation of interferon-γ, interleukin 5, and Treg cytokines in asthma patient peripheral blood mononuclear cells by ganoderic acid B. Int. Urogynecol J. 33, 1231–1240. 10.1002/ptr.7266 35112740

[B111] Liu J. Wu Z. Liu Y. Zhan Z. Yang L. Wang C. (2022). ROS-responsive liposomes as an inhaled drug delivery nanoplatform for idiopathic pulmonary fibrosis treatment via Nrf2 signaling. J. Nanobiotechnology 20, 213. 10.1186/s12951-022-01435-4 35524280 PMC9074278

[B112] Liu X. Zhou W. Zhang X. Lu P. Du Q. Tao L. (2016). Dimethyl fumarate ameliorates dextran sulfate sodium-induced murine experimental colitis by activating Nrf2 and suppressing NLRP3 inflammasome activation. Biochem. Pharmacol. 112, 37–49. 10.1016/j.bcp.2016.05.002 27184504

[B113] Liu Y. Qiu J. Wang Z. You W. Wu L. Ji C. (2015). Dimethylfumarate alleviates early brain injury and secondary cognitive deficits after experimental subarachnoid hemorrhage via activation of Keap1-Nrf2-ARE system. J. Neurosurg. 123, 915–923. 10.3171/2014.11.JNS132348 25614941

[B114] Loewe R. Valero T. Kremling S. Pratscher B. Kunstfeld R. Pehamberger H. (2006). Dimethylfumarate impairs melanoma growth and metastasis. Cancer Res. 66, 11888–11896. 10.1158/0008-5472.CAN-06-2397 17178886

[B115] Lone A. Behl T. Kumar A. Makkar R. Priya N. Redhu S. (2020). Renoprotective potential of dimethyl fumarate in streptozotocin induced diabetic nephropathy in Wistar rats. Indian J. Pharmacol. 45, 100237–100323. 10.1016/j.obmed.2020.100237

[B116] Longbrake E. E. Ramsbottom M. J. Cantoni C. Ghezzi L. Cross A. H. Piccio L. (2016). Dimethyl fumarate selectively reduces memory T cells in multiple sclerosis patients. Mult. Scler. J. 22, 1061–1070. 10.1177/1352458515608961 PMC4829494 26459150

[B117] Lu T. Sun X. Li Y. Chai Q. Wang X-L. Lee H-C. (2017). Role of Nrf2 signaling in the regulation of vascular BK channel β1 subunit expression and BK channel function in high-fat diet–induced diabetic mice. Diabetes 66, 2681–2690. 10.2337/db17-0181 28465407 PMC5606315

[B118] Luo M. Sun Q. Zhao H. Tao J. Yan D. (2019). The effects of dimethyl fumarate on atherosclerosis in the apolipoprotein E-deficient mouse model with streptozotocin-induced hyperglycemia mediated by the nuclear factor erythroid 2-related factor 2/antioxidant response element (Nrf2/ARE) signaling pathway. Med. Sci. Monit. 25, 7966–7975. 10.12659/MSM.918951 31645538 PMC6824188

[B119] Majkutewicz I. Kurowska E. Podlacha M. Myślińska D. Grembecka B. Ruciński J. (2018). Age-dependent effects of dimethyl fumarate on cognitive and neuropathological features in the streptozotocin-induced rat model of Alzheimer’s disease. Brain Res. 1686, 19–33. 10.1016/j.brainres.2018.02.016 29453958

[B120] Ma N. Wu Y. Xie F. Du K. Wang Y. Shi L. (2017). Dimethyl fumarate reduces the risk of mycotoxins via improving intestinal barrier and microbiota. Oncotarget 8, 44625–44638. 10.18632/oncotarget.17886 28574825 PMC5546506

[B121] Majkutewicz I. Kurowska E. Podlacha M. Myślińska D. Grembecka B. Ruciński J. (2016). Dimethyl fumarate attenuates intracerebroventricular streptozotocin-induced spatial memory impairment and hippocampal neurodegeneration in rats. Behav. Brain Res. 308, 24–37. 10.1016/j.bbr.2016.04.012 27083302

[B122] Manai F. Govoni S. Amadio M. (2022). The challenge of dimethyl fumarate repurposing in eye pathologies. Cells 11, 4061. 10.3390/cells11244061 36552824 PMC9777082

[B123] Mauro D. Manou-Stathopoulou S. Rivellese F. Sciacca E. Goldmann K. Tsang V. (2023). UBE2L3 regulates TLR7-induced B cell autoreactivity in Systemic Lupus Erythematosus. J. Autoimmun. 136, 103023. 10.1016/j.jaut.2023.103023 37001433

[B124] McGuire V. A. Ruiz-Zorrilla Diez T. Emmerich C. H. Strickson S. Ritorto M. S. Sutavani R. V. (2016). Dimethyl fumarate blocks pro-inflammatory cytokine production via inhibition of TLR induced M1 and K63 ubiquitin chain formation. Sci. Rep. 6, 31159. 10.1038/srep31159 27498693 PMC4976367

[B125] Meili-Butz S. Niermann T. Fasler-Kan E. Barbosa V. Butz N. John D. (2008). Dimethyl fumarate, a small molecule drug for psoriasis, inhibits Nuclear Factor-kappaB and reduces myocardial infarct size in rats. Eur. J. Pharmacol. 586, 251–258. 10.1016/j.ejphar.2008.02.038 18405893

[B126] Meissner M. Valesky E. M. Kippenberger S. Kaufmann R. (2012). Dimethyl fumarate - only an anti-psoriatic medication? J. Dtsch. Dermatol Ges. 10, 793–801. 10.1111/j.1610-0387.2012.07996.x 22897153

[B127] Mi Y. Xie X. Bao Z. Xiong X. Wang X. Zhang H. (2023). Dimethyl fumarate protects against intestine damage in necrotizing enterocolitis by inhibiting the Toll-like receptor (TLR) inflammatory signaling pathway. Tissue Cell 81, 102003. 10.1016/j.tice.2022.102003 36682224

[B128] Milad S. Jangholi E. Seyed F. M. Rostami M. Bereimipour A. Majidi S. (2022). Effects of dimethyl fumarate on the karnofsky performance status and serum S100β level in newly glioblastoma patients: A randomized, phase-II, placebo, triple blinded, controlled trial: Effect of DMF on the serum S100β level and KPS score of GBM patients. Galen. Med. J. 10, 1–10. 10.31661/gmj.v11i.1897 PMC9616682 36340958

[B129] Miljković D. Blaževski J. Petković F. Djedović N. Momčilović M. Stanisavljević S. (2015). A comparative analysis of multiple sclerosis-relevant anti-inflammatory properties of ethyl pyruvate and dimethyl fumarate. J. Immunol. 194, 2493–2503. 10.4049/jimmunol.1402302 25681336

[B130] Mohle L. Brackhan M. Bascuñana P. Pahnke J. (2021). Dimethyl fumarate does not mitigate cognitive decline and β-amyloidosis in female APPPS1 mice. Brain Res. 1768, 147579. 10.1016/j.brainres.2021.147579 34233173

[B131] Montes Diaz G. Fraussen J. Van Wijmeersch B. Hupperts R. Somers V. (2018). Dimethyl fumarate induces a persistent change in the composition of the innate and adaptive immune system in multiple sclerosis patients. Sci. Rep. 8, 8194. 10.1038/s41598-018-26519-w 29844361 PMC5974280

[B132] Mori S. Kurimoto T. Maeda H. Nakamura M. (2020). Dimethyl fumarate promotes the survival of retinal ganglion cells after optic nerve injury, possibly through the Nrf2/HO-1 pathway. Int. J. Mol. Sci. 22, 297. 10.3390/ijms22010297 33396673 PMC7795407

[B133] Morris G. Sominsky L. Walder K. R. Berk M. Marx W. Carvalho A. F. (2022). Inflammation and nitro-oxidative stress as drivers of endocannabinoid system aberrations in mood disorders and schizophrenia. Mol. Neurobiol. 59, 3485–3503. 10.1007/s12035-022-02800-y 35347586

[B134] Mouton A. J. Flynn E. R. Moak S. P. Aitken N. M. Omoto A. C. M. Li X. (2021). Dimethyl fumarate preserves left ventricular infarct integrity following myocardial infarction via modulation of cardiac macrophage and fibroblast oxidative metabolism. J. Mol. Cell Cardiol. 158, 38–48. 10.1016/j.yjmcc.2021.05.008 34023353 PMC8522337

[B135] Mrowietz U. Szepietowski J. C. Loewe R. van de Kerkhof P. Lamarca R. Ocker W. G. (2016). Efficacy and safety of LAS41008 (dimethyl fumarate) in adults with moderate-to-severe chronic plaque psoriasis: A randomized, double-blind, Fumaderm® - and placebo-controlled trial (BRIDGE). Br. J. Dermatol 176, 615–623. 10.1111/bjd.14947 27515097

[B136] Nachliely M. Trachtenberg A. Khalfin B. Nalbandyan K. Cohen-Lahav M. Yasuda K. (2019). Dimethyl fumarate and vitamin D derivatives cooperatively enhance VDR and Nrf2 signaling in differentiating AML cells *in vitro* and inhibit leukemia progression in a xenograft mouse model. J. Steroid Biochem. Mol. Biol. 188, 8–16. 10.1016/j.jsbmb.2018.11.017 30508646

[B137] Naismith R. T. Wundes A. Ziemssen T. Jasinska E. Freedman M. S. Lembo A. J. (2020). Diroximel fumarate demonstrates an improved gastrointestinal tolerability profile compared with dimethyl fumarate in patients with relapsing–remitting multiple sclerosis: Results from the randomized, double-blind, phase III EVOLVE-MS-2 study. CNS Drugs 34, 185–196. 10.1007/s40263-020-00700-0 31953790 PMC7018784

[B138] Ni H. M. Boggess N. McGill M. R. Lebofsky M. Borude P. Apte U. (2012). Liver-specific loss of Atg5 causes persistent activation of Nrf2 and protects against acetaminophen-induced liver injury. Toxicol. Sci. 127, 438–450. 10.1093/toxsci/kfs133 22491424 PMC3355320

[B139] Ni H. M. Woolbright B. L. Williams J. Copple B. Cui W. Luyendyk J. P. (2014). Nrf2 promotes the development of fibrosis and tumorigenesis in mice with defective hepatic autophagy. J. Hepatol. 61, 617–625. 10.1016/j.jhep.2014.04.043 24815875 PMC4143992

[B140] NicolayJ. P.MelchersS.AlbrechtJ. D.AssafC.DippelE.StadlerR. (2023). Dimethyl fumarate treatment in relapsed and refractory cutaneous T cell lymphoma - a multicenter phase II study. Blood blood, 2022018669. 10.1182/blood.2022018669 37217183

[B141] Nicolay J. P. Müller-Decker K. Anne Pauline S. Brechmann M. Mobs M. Géraud C. (2016). Dimethyl fumarate restores apoptosis sensitivity and inhibits tumor growth and metastasis in CTCL by targeting NF-κB. Blood 128, 805–815. 10.1182/blood-2016-01-694117 27268084 PMC5026464

[B142] Nograles K. E. Krueger J. G. (2011). Anti-cytokine therapies for psoriasis. Exp. Cell Res. 317, 1293–1300. 10.1016/j.yexcr.2011.01.024 21300061

[B143] Nour O. A. Shehatou G. S. G. Rahim M. A. El-Awady M. Suddek G. (2017). Antioxidant and anti-inflammatory effects of dimethyl fumarate in hypercholesterolemic rabbits. Egypt J. Basic Appl. Sci. 4, 153–159. 10.1016/j.ejbas.2017.07.003

[B144] Offermanns S. (2017). Hydroxy-carboxylic acid receptor actions in metabolism. Trends Endocrinol. Metab. 28, 227–236. 10.1016/j.tem.2016.11.007 28087125

[B145] Ogawa E. Sato Y. Minagawa A. Okuyama R. (2017). Pathogenesis of psoriasis and development of treatment. J. Dermatol 45, 264–272. 10.1111/1346-8138.14139 29226422

[B146] Olagnier D. Farahani E. Thyrsted J. Blay-Cadanet J. Herengt A. Idorn M. (2020). SARS-CoV2-mediated suppression of NRF2-signaling reveals potent antiviral and anti-inflammatory activity of 4-octyl-itaconate and dimethyl fumarate. Nat. Commun. 11, 4938. 10.1038/s41467-020-18764-3 33009401 PMC7532469

[B147] Palte M. J. Wehr A. Tawa M. Perkin K. Leigh-Pemberton R. Hanna J. (2019). Improving the gastrointestinal tolerability of fumaric acid esters: Early findings on gastrointestinal events with diroximel fumarate in patients with relapsing-remitting multiple sclerosis from the phase 3, open-label EVOLVE-MS-1 study. Adv. Ther. 36, 3154–3165. 10.1007/s12325-019-01085-3 31538304 PMC6822793

[B148] Paolinelli M. Diotallevi F. Martina E. Radi G. Bianchelli T. Giacchetti A. (2022). New and old horizons for an ancient drug: Pharmacokinetics, pharmacodynamics, and clinical perspectives of dimethyl fumarate. Pharmaceutics 14, 2732. 10.3390/pharmaceutics14122732 36559226 PMC9788528

[B149] Parodi B. Rossi S. Morando S. Cordano C. Bragoni A. Motta C. (2015). Fumarates modulate microglia activation through a novel HCAR2 signaling pathway and rescue synaptic dysregulation in inflamed CNS. Acta Neuropathol. 130, 279–295. 10.1007/s00401-015-1422-3 25920452 PMC4503882

[B150] Parodi B. Sanna A. Cedola A. Uccelli A. Kerlero de Rosbo N. (2021). Hydroxycarboxylic acid receptor 2, a pleiotropically linked receptor for the multiple sclerosis drug, monomethyl fumarate. Possible implications for the inflammatory response. Front. Immunol. 12, 655212. 10.3389/fimmu.2021.655212 34084164 PMC8167049

[B151] Peng H. Guerau-de-Arellano M. Mehta V. B. Yang Y. Huss D. J. Papenfuss T. L. (2012). Dimethyl fumarate inhibits dendritic cell maturation via nuclear factor κB (NF-κB) and extracellular signal-regulated kinase 1 and 2 (ERK1/2) and mitogen stress-activated kinase 1 (MSK1) signaling. J. Biol. Chem. 287, 28017–28026. 10.1074/jbc.M112.383380 22733812 PMC3431702

[B152] Pereira M. A. Barnes L. H. Rassman V. L. Kelloff G. V. Steele V. E. (1994). Use of azoxymethane-induced foci of aberrant crypts in rat colon to identify potential cancer chemopreventive agents. Carcinogenesis 15, 1049–1054. 10.1093/carcin/15.5.1049 8200067

[B153] Pinto B. F. Ribeiro L. N. B. da Silva G. B. R. F. Freitas C. S. Kraemer L. Oliveira F. M. S. (2022). Inhalation of dimethyl fumarate-encapsulated solid lipid nanoparticles attenuate clinical signs of experimental autoimmune encephalomyelitis and pulmonary inflammatory dysfunction in mice. Clin. Sci. 136, 81–101. 10.1042/CS20210792 34904644

[B154] Preiningerova J. L. Jiraskova Zakostelska Z. Srinivasan A. Ticha V. Kovarova I. Kleinova P. (2022). Multiple sclerosis and microbiome. Biomolecules 12, 433. 10.3390/biom12030433 35327624 PMC8946130

[B155] Qi D. Chen P. Bao H. Zhang L. Sun K. Song S. (2023). Dimethyl fumarate protects against hepatic ischemia-reperfusion injury by alleviating ferroptosis via the NRF2/SLC7A11/HO-1 axis. Cell cycle 22, 818–828. 10.1080/15384101.2022.2155016 36482709 PMC10026899

[B156] Ragab D. Abdallah D. M. El-Abhar H. S. (2020). The dual reno- and neuro-protective effects of dimethyl fumarate against uremic encephalopathy in a renal ischemia/reperfusion model. Pharmacol. Rep. 72, 969–983. 10.1007/s43440-020-00076-4 32141026

[B157] Ranea‐Robles P. Launay N. Ruiz M. Calingasan N. Y. Dumont M. Naudí A. (2018). Aberrant regulation of the GSK ‐3β/NRF 2 axis unveils a novel therapy for adrenoleukodystrophy. EMBO Mol. Med. 10, e8604. 10.15252/emmm.201708604 29997171 PMC6079538

[B158] Rao R. Rivenson A. Kelloff G. J. Reddy S. (1995). Chemoprevention of azoxymethane-induced colon cancer by ascorbylpalmitate, carbenoxolone, dimethylfumarate and p-methoxyphenol in male F344 rats. Anticancer Res. 15, 1199–1204. 7653999

[B159] Reich K. Thaci D. Mrowietz U. Kamps A. Neureither M. Luger T. (2009). Efficacy and safety of fumaric acid esters in the long-term treatment of psoriasis-a retrospective study (FUTURE). J. Dtsch. Dermatol Ges. 7, 603–611. 10.1111/j.1610-0387.2009.07120.x 19459898

[B160] Rojo A. I. Pajares M. García-Yagüe A. J. Buendia I. Van Leuven F. Yamamoto M. (2018). Deficiency in the transcription factor NRF2 worsens inflammatory parameters in a mouse model with combined tauopathy and amyloidopathy. Redox Biol. 18, 173–180. 10.1016/j.redox.2018.07.006 30029164 PMC6052199

[B161] Rojo de la Vega M. Chapman E. Zhang D. D. (2018). NRF2 and the hallmarks of cancer. Cancer Cell 34, 21–43. 10.1016/j.ccell.2018.03.022 29731393 PMC6039250

[B162] Rupp T. Debasly S. Genest L. Froget G. Castagné V. (2022). Therapeutic potential of fingolimod and dimethyl fumarate in non-small cell lung cancer preclinical models. Int. J. Mol. Sci. 23, 8192. 10.3390/ijms23158192 35897763 PMC9330228

[B163] Scannevin R. Chollate S. Jung M. Shackett M. Patel H. Bista P. (2012). Fumarates promote cytoprotection of central nervous system cells against oxidative stress via the nuclear factor (Erythroid-Derived 2)-like 2 pathway. J. Pharmacol. Exp. Ther. 341, 274–284. 10.1124/jpet.111.190132 22267202

[B164] Sachinvala N. D. Teramoto N. Stergiou A. (2020). Proposed neuroimmune roles of dimethyl fumarate, bupropion, S-adenosylmethionine, and vitamin D3 in affording a chronically ill patient sustained relief from inflammation and major depression. Brain Sci. 10, 600. 10.3390/brainsci10090600 32878267 PMC7563300

[B165] Safari A. Badeli-Sarkala H. Namavar M. R. Kargar-Abarghouei E. Anssari N. Izadi S. (2019). Neuroprotective effect of dimethyl fumarate in stroke: The role of nuclear factor erythroid 2-related factor 2. Iran. J. Neurol. 18, 108–113. 10.18502/ijnl.v18i3.1633 31749931 PMC6858603

[B166] Saidu N. E. B. Kavian N. Leroy K. Jacob C. Nicco C. Batteux F. (2019). Dimethyl fumarate, a two-edged drug: Current status and future directions. Med. Res. Rev. 39, 1923–1952. 10.1002/med.21567 30756407

[B167] Saidu N. E. B. Noé G. Cerles O. Cabel L. Kavian-Tessler N. Chouzenoux S. (2017). Dimethyl fumarate controls the NRF2/DJ-1 Axis in cancer cells: Therapeutic applications. Mol. Cancer Ther. 16, 529–539. 10.1158/1535-7163.MCT-16-0405 28069874

[B168] Sánchez-García F. J. Pérez-Hernández C. A. Rodríguez-Murillo M. Moreno-Altamirano M. M. B. (2021). The role of tricarboxylic acid cycle metabolites in viral infections. Front. Cell Infect. Microbiol. 11, 725043. 10.3389/fcimb.2021.725043 34595133 PMC8476952

[B169] Schilling S. Goelz S. Linker R. Luehder F. Gold R. (2006). Fumaric acid esters are effective in chronic experimental autoimmune encephalomyelitis and suppress macrophage infiltration. Clin. Exp. Immunol. 145, 101–107. 10.1111/j.1365-2249.2006.03094.x 16792679 PMC1942010

[B170] Schimrigk S. Brune N. Hellwig K. Lukas C. Bellenberg B. Rieks M. (2006). Oral fumaric acid esters for the treatment of active multiple sclerosis: An open-label, baseline-controlled pilot study. Eur. J. Neurol. 13, 604–610. 10.1111/j.1468-1331.2006.01292.x 16796584

[B171] Schmieder A. Poppe M. Hametner C. Meyer-Schraml H. Schaarschmidt M-L. Findeisen P. (2015). Impact of fumaric acid esters on cardiovascular risk factors and depression in psoriasis: A prospective pilot study. Arch. Dermatol Res. 307, 413–424. 10.1007/s00403-015-1541-7 25648959

[B172] Schweckendiek W. (1959). Treatment of psoriasis vulgaris. Med. Monatsschr 13, 103–104. 13643669

[B173] Scuderi S. A. Ardizzone A. Paterniti I. Esposito E. Campolo M. (2020). Antioxidant and anti-inflammatory effect of Nrf2 inducer dimethyl fumarate in neurodegenerative diseases. Antioxidants (Basel) 9, 630. 10.3390/antiox9070630 32708926 PMC7402174

[B174] Seidel P. Roth M. (2013). Anti-inflammatory dimethylfumarate: A potential new therapy for asthma? Mediat. Inflamm. 2013, 875403. 10.1155/2013/875403 PMC3625606 23606796

[B175] Seminotti B. Grings M. Tucci P. Leipnitz G. Saso L. (2021). Nuclear factor erythroid-2-related factor 2 signaling in the neuropathophysiology of inherited metabolic disorders. Front. Cell Neurosci. 15, 785057. 10.3389/fncel.2021.785057 34955754 PMC8693715

[B176] Shafer D. Chen Z. J. Harris T. D. Beth Tombes M. Shrader E. Strickler K. (2017). Phase I trial of dimethyl fumarate, temozolomide, and radiation therapy in glioblastoma. Neurooncol Adv. 2, vdz052. 10.1093/noajnl/vdz052 PMC7212848 32642720

[B177] Sheikh S. I. Nestorov I. Russell H. O'Gorman J. Huang R. Milne G. L. (2013). Tolerability and pharmacokinetics of delayed-release dimethyl fumarate administered with and without aspirin in healthy volunteers. Clin. Ther. 35, 1582–1594. 10.1016/j.clinthera.2013.08.009 24139424

[B178] Shi F. L. Yuan L. S. Wong T. S. Li Q. Li Y. P. Xu R. (2023). Dimethyl fumarate inhibits necroptosis and alleviates systemic inflammatory response syndrome by blocking the RIPK1-RIPK3-MLKL axis. Pharmacol. Res. 189, 106697. 10.1016/j.phrs.2023.106697 36796462

[B179] Sicurella M. Pula W. Musiał K. Cieślik-Boczula K. Sguizzato M. Bondi A. (2023). Ethosomal gel for topical administration of dimethyl fumarate in the treatment of HSV-1 infections. Int. J. Mol. Sci. 24, 4133. 10.3390/ijms24044133 36835541 PMC9967198

[B180] Simpson-Yap S. De Brouwer E. Kalincik T. Rijke N. Hillert J. A. Walton C. (2021). Associations of disease-modifying therapies with COVID-19 severity in multiple sclerosis. Neurology 97, e1870–e1885. 10.1212/WNL.0000000000012753 34610987 PMC8601210

[B181] Smith T. E. Madhavan M. Gratch D. Patel A. Saha V. Sammarco C. (2022). Risk of COVID-19 infection and severe disease in MS patients on different disease-modifying therapies. Mult. Scler. Relat. Disord. 60, 103735. 10.1016/j.msard.2022.103735 35398713 PMC8915504

[B182] Solana-Manrique C. Sanz F. J. Ripollés E. Bañó M. C. Torres J. Muñoz-Soriano V. (2020). Enhanced activity of glycolytic enzymes in Drosophila and human cell models of Parkinson’s disease based on DJ-1 deficiency. Free Radic. Biol. Med. 158, 137–148. 10.1016/j.freeradbiomed.2020.06.036 32726690

[B183] Stamatellos V. Siafis S. Papazisis G. (2021). Disease-modifying agents for multiple sclerosis and the risk for reporting cancer: A disproportionality analysis using the US food and drug administration adverse event reporting system database. Br. J. Clin. Pharmacol. 87, 4769–4779. 10.1111/bcp.14916 33998034

[B184] Storm-Larsen C. Myhr K-M. Farbu E. Midgard R. Nyquist K. B. Broch L. (2019). Gut microbiota composition during a 12-week intervention with delayed-release dimethyl fumarate in multiple sclerosis – A pilot trial. Mult. Scler. J. Exp. Transl. Clin. 5, 2055217319888767. 10.1177/2055217319888767 31798939 PMC6859687

[B185] Sun X. Suo X. Xia X. Yu C. Dou Y. (2022a). Dimethyl fumarate is a potential therapeutic option for Alzheimer’s disease. J. Alzheimers Dis. 85, 443–456. 10.3233/JAD-215074 34842188

[B186] Sun Z. Liu X. Liu Y. Zhao X. Zang X. Wang F. (2022b). Immunosuppressive effects of dimethyl fumarate on dendritic cell maturation and migration: A potent protector for coronary heart disease. Immunopharmacol. Immunotoxicol. 44, 178–185. 10.1080/08923973.2021.2025245 35016591

[B187] Takasu C. Vaziri N. D. Li S. Robles L. Vo K. Takasu M. (2017). Treatment with dimethyl fumarate ameliorates liver ischemia/reperfusion injury. World J. Gastroenterol. 23, 4508–4516. 10.3748/wjg.v23.i25.4508 28740339 PMC5504366

[B188] Takeda T. Tsubaki M. Asano R. Itoh T. Imano M. Takao S. (2020). Dimethyl fumarate suppresses metastasis and growth of melanoma cells by inhibiting the nuclear translocation of NF-κB. J. Dermatol Sci. 99, 168–176. 10.1016/j.jdermsci.2020.07.004 32693971

[B189] Tastan B. Arioz B. I. Genc S. (2022). Targeting NLRP3 inflammasome with Nrf2 inducers in central nervous system disorders. Front. Immunol. 13, 865772. 10.3389/fimmu.2022.865772 35418995 PMC8995746

[B190] Teleanu D. M. Niculescu A. G. Lungu II Radu C. I. Vladâcenco O. Roza E. (2022). An overview of oxidative stress, neuroinflammation, and neurodegenerative diseases. Int. J. Mol. Sci. 23, 5938. 10.3390/ijms23115938 35682615 PMC9180653

[B191] Tian K. Yang Y. Zhou K. Deng N. Tian Z. Wu Z. (2023). The role of ROS-induced pyroptosis in CVD. Front. Cardiovasc Med. 10, 1116509. 10.3389/fcvm.2023.1116509 36873396 PMC9978107

[B192] Timpani C. A. Rybalka E. (2020). Calming the (cytokine) storm: Dimethyl fumarate as a therapeutic candidate for COVID-19. Pharm. (Basel) 14, 15. 10.3390/ph14010015 PMC7824470 33375288

[B193] Todorich B. Zhang X. Connor J. R. (2011). H-ferritin is the major source of iron for oligodendrocytes. Glia 59, 927–935. 10.1002/glia.21164 21446040

[B194] Treumer F. Zhu K. Gläser R. Mrowietz U. (2003). Dimethyl fumarate is a potent inducer of apoptosis in human T cells. J. Investigative Dermatology 121, 1383–1388. 10.1111/j.1523-1747.2003.12605.x 14675187

[B195] Tsianakas A. Herzog S. Landmann A. Patsinakidis N. Perusquía Ortiz A. M. Bonsmann G. (2014). Successful treatment of discoid lupus erythematosus with fumaric acid esters. J. Am. Acad. Dermatol 71, e15–e17. e15–e17. 10.1016/j.jaad.2013.12.004 24947703

[B196] Valero T. Steele S. Neumüller K. Bracher A. Niederleithner H. Pehamberger H. (2010). Combination of dacarbazine and dimethylfumarate efficiently reduces melanoma lymph node metastasis. J. Invest Dermatol 130, 1087–1094. 10.1038/jid.2009.368 19940857

[B197] Van Dingenen J. Pieters L. Van Nuffel E. Lefebvre R. A. (2019). Hemin reduces postoperative ileus in a heme oxygenase 1‐dependent manner while dimethyl fumarate does without heme oxygenase 1‐induction. Neurogastroenterol. Motil. 32, e13624. 10.1111/nmo.13624 31121086

[B198] Vucic S. Henderson R. D. Mathers S. Needham M. Schultz D. Kiernan M. C. (2021). Safety and efficacy of dimethyl fumarate in ALS: Randomised controlled study. Ann. Clin. Transl. Neurol. 8, 1991–1999. –1999. 10.1002/acn3.51446 34477330 PMC8528453

[B199] Wang X-L. Zhu Q-Q. Simayi A. Xu G-P. (2023). Nrf2 protects against myocardial ischemia-reperfusion injury in diabetic rats by inhibiting Drp1-mediated mitochondrial fission. Open Med. (Wars) 18, 20230711. 10.1515/med-2023-0711 37333454 PMC10276614

[B200] Werdenberg D. Joshi R. Wolffram S. Merkle H. P. Langguth P. (2003). Presystemic metabolism and intestinal absorption of antipsoriatic fumaric acid esters. Biopharm. Drug Dispos. 24, 259–273. 10.1002/bdd.364 12973823

[B201] Wilms H. Sievers J. Rickert U. Rostami-Yazdi M. Mrowietz U. Lucius R. (2010). Dimethylfumarate inhibits microglial and astrocytic inflammation by suppressing the synthesis of nitric oxide, IL-1beta, TNF-alpha and IL-6 in an *in-vitro* model of brain inflammation. J. Neuroinflammation 7, 30. 10.1186/1742-2094-7-30 20482831 PMC2880998

[B202] Wollina U. (2011). Fumaric acid esters in dermatology. Indian Dermatol Online J. 2, 111–119. 10.4103/2229-5178.86007 23130241 PMC3481830

[B203] Wrona D. Majkutewicz I. Świątek G. Dunacka J. Grembecka B. Glac W. (2022). Dimethyl fumarate as the peripheral blood inflammatory mediators inhibitor in prevention of streptozotocin-induced neuroinflammation in aged rats. J. Inflamm. Res. 15, 33–52. 10.2147/JIR.S342280 35027835 PMC8749052

[B204] Yao Y. Miao W. Liu Z. Han W. Shi K. Shen Y. (2016). Dimethyl fumarate and monomethyl fumarate promote post-ischemic recovery in mice. Transl. Stroke Res. 7, 535–547. 10.1007/s12975-016-0496-0 27614618 PMC5065588

[B205] Yeung F. Hoberg J. E. Ramsey C. S. Keller M. D. Jones D. R. Frye R. A. (2004). Modulation of NF-kappaB-dependent transcription and cell survival by the SIRT1 deacetylase. EMBO J. 23, 2369–2380. 10.1038/sj.emboj.7600244 15152190 PMC423286

[B206] You Y. Barnett M. Yiannikas C. Parratt J. Matthews J. G. Graham S. L. (2021). Interferon-β is less effective than other drugs in controlling the rate of retinal ganglion cell loss in MS. Neurol. Neuroimmunol. Neuroinflamm 8, e971. 10.1212/NXI.0000000000000971 33597189 PMC8105907

[B207] Yu J. Li Y. Li Z. Li H. Chen Y. Chen X. (2021). Subconjunctival injections of dimethyl fumarate inhibit lymphangiogenesis and allograft rejection in the rat cornea. Int. Immunopharmacol. 96, 107580. 10.1016/j.intimp.2021.107580 33823430

[B208] Zhao H. Eguchi S. Alam A. Ma D. (2017). The role of nuclear factor-erythroid 2 related factor 2 (Nrf-2) in the protection against lung injury. Am. J. Physiol. Lung Cell Mol. Physiol. 312, L155-L162–L162. 10.1152/ajplung.00449.2016 27864288

[B209] Zhao J. Cheng Z. Quan X. Xie Z. Zhang L. Ding Z. (2020). Dimethyl fumarate protects cardiomyocytes against oxygen-glucose deprivation/reperfusion (OGD/R)-induced inflammatory response and damages via inhibition of Egr-1. Int. Immunopharmacol. 86, 106733. 10.1016/j.intimp.2020.106733 32645629

[B210] Zhao X. Sun G. Zhang J. Ting S-M. Gonzales N. Aronowski J. (2015). Dimethyl fumarate protects brain from damage produced by intracerebral hemorrhage by mechanism involving Nrf2. Stroke 46, 1923–1928. –1928. 10.1161/STROKEAHA.115.009398 25977275 PMC4480061

[B211] Zhen X. Jindong L. Yang Z. Yashi R. Wei G. Wei J. (2021). Activation of Nrf2 pathway by dimethyl fumarate attenuates renal ischemia-reperfusion injury. Transpl. Proc. 53, 2133–2139. 10.1016/j.transproceed.2021.07.017 34426023

[B212] Zhou S. Ye W. Shao Q. Zhang M. Liang J. (2013). Nrf2 is a potential therapeutic target in radioresistance in human cancer. Crit. Rev. Oncol. Hematol. 88, 706–715. 10.1016/j.critrevonc.2013.09.001 24126138

[B213] Zhou Y. Zhang F. Jiang H. Xu D. Deng D. (2021). Fumaric acid and succinic acid treat gestational hypertension by downregulating the expression of KCNMB1 and TET1. Exp. Ther. Med. 22, 1072. 10.3892/etm.2021.10506 34447465 PMC8355717

[B214] Zyla K. Larabee C. M. Georgescu C. Berkley C. Reyna T. Plafker S. M. (2019). Dimethyl fumarate mitigates optic neuritis. Mol. Vis. 25, 446–461. 31523122 PMC6707756

