# Supplementary material for: Novel potential pharmacological applications of dimethyl fumarate—an overview and update
Source: Front Pharmacol. 2023 Sep 7;14:1264842. doi: 10.3389/fphar.2023.1264842 (PMC10512734; doi:10.3389/fphar.2023.1264842)
Supplement: Supplementary file 1 [file Table1.DOCX]

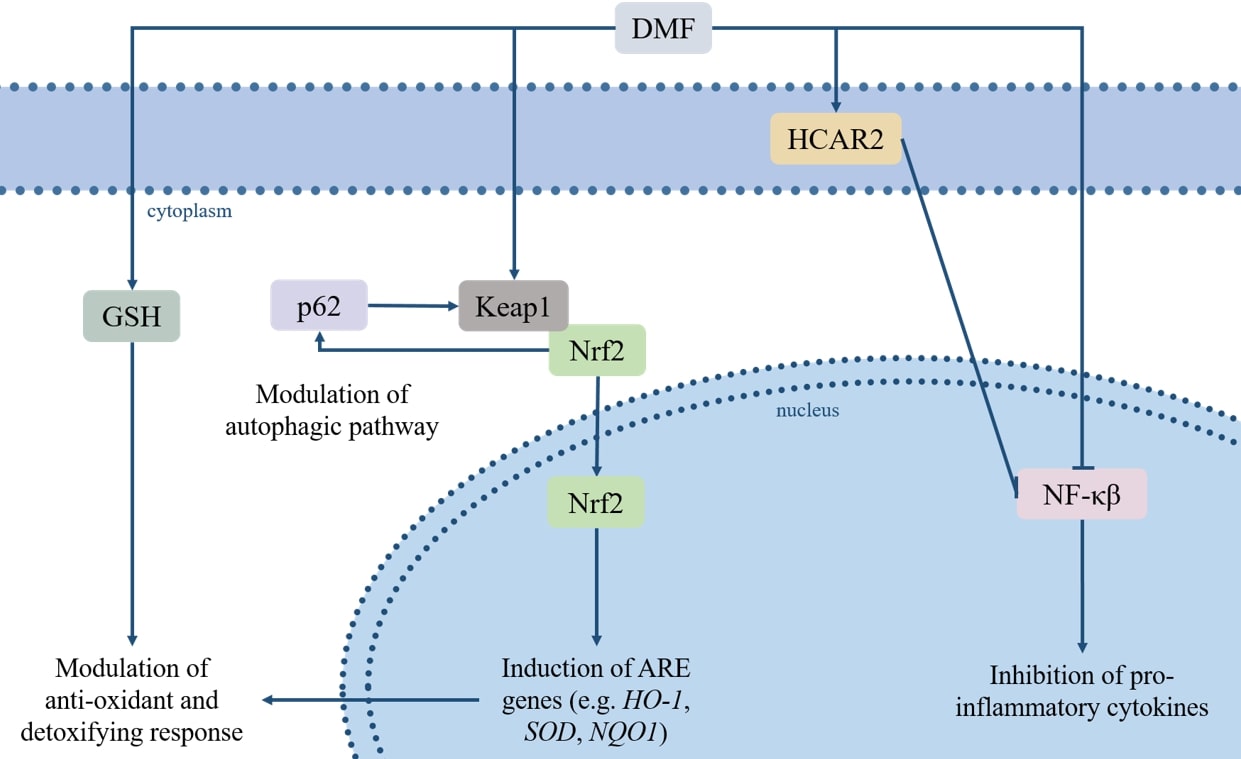
Figure 1: DMF's immunomodulatory and antioxidative effects rely on its regulation of several pathways, among which the Keap1/Nrf2/ARE and NF-κB pathways, the modulation of GSH levels, its agonism of HCAR2 and its effects on the autophagic system.Based on these properties, DMF has sparked interest due to its potential repurposing for a variety of pathologies characterized or aggravated by inflammatory processes and oxidative stress. Abbreviations: dimethyl fumarate (DMF), glutathione (GSH), Hydroxycarboxylic Acid Receptor 2 (HCAR2), sequestrome 1 (p62/SQSTM1), Kelch-like ECH-associated protein (Keap1), nuclear factor erythroid 2-related factor 2 (Nrf2), nuclear factor kappa-light-chain-enhancer of activated B cells (NF-κB), *hemoxygenase-1* (*HO1*), *quinoline oxidoreductase-1* (*NQO1*), *superoxide dismutase* (*SOD*).


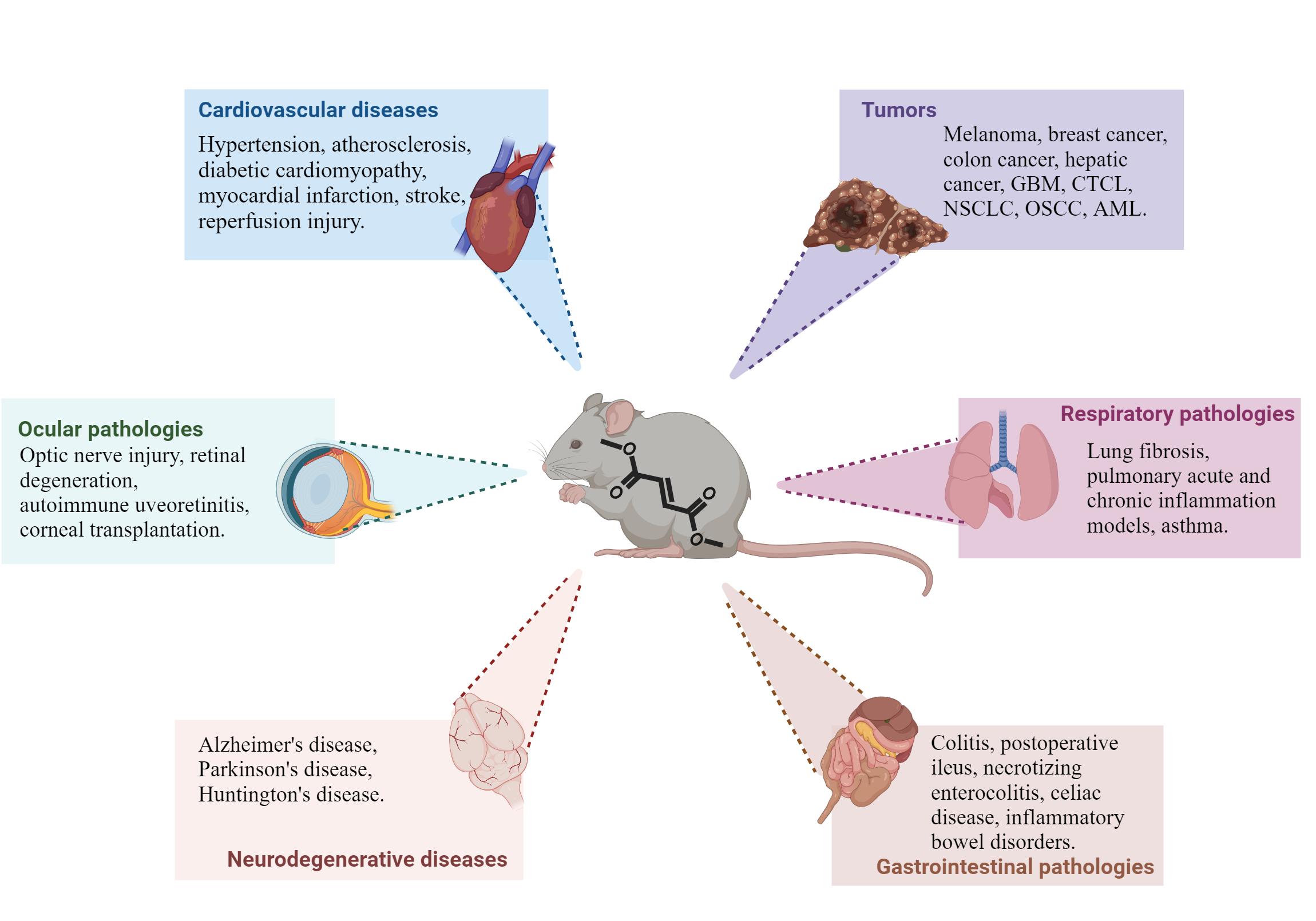
Figure 2: Main pathological contexts reporting protective or beneficial effects of DMF treatment. Recent studies demonstrate DMF’s efficacy in several *in vivo* models of different pathologies; in particular, preclinical data is available on different cardiovascular, neurodegenerative, ocular and gastrointestinal diseases, as well as tumors, as shown in the picture. DMF’s effect is also being investigated in other pathologies outside of these main contexts (see text for additional information). Abbreviations: GBM: glioblastoma multiforme; CTCL: cutaneous T-cell lymphoma; NSCLC: non-small cells lung cancer; OSCC: oral squamous cell carcinoma; AML: acute myeloid leukemia.
